# NLRP3 Inflammasome-Dependent Increases in High Mobility Group Box 1 Involved in the Cognitive Dysfunction Caused by Tau-Overexpression

**DOI:** 10.3389/fnagi.2021.721474

**Published:** 2021-09-03

**Authors:** Yan Zhao, Si-Wei Tan, Zhi-Zhong Huang, Fa-Bo Shan, Ping Li, Ya-Lei Ning, Shi-Yang Ye, Zi-Ai Zhao, Hao Du, Ren-Ping Xiong, Nan Yang, Yan Peng, Xing Chen, Yuan-Guo Zhou

**Affiliations:** ^1^Department of Army Occupational Disease, State Key Laboratory of Trauma, Burns and Combined Injury, Research Institute of Surgery, Daping Hospital, Army Medical University, Chongqing, China; ^2^Institute of Brain and Intelligence, Army Medical University, Chongqing, China; ^3^Department of Neurology, General Hospital of Northern Theater Command, Shenyang, China

**Keywords:** traumatic brain injury, high mobility group box-1, tau, NLRP3 inflammasome, spatial memory, hippocampus

## Abstract

Tau hyperphosphorylation is a characteristic alteration present in a range of neurological conditions, such as traumatic brain injury (TBI) and neurodegenerative diseases. Treatments targeting high-mobility group box protein 1 (HMGB1) induce neuroprotective effects in these neuropathologic conditions. However, little is known about the interactions between hyperphosphorylated tau and HMGB1 in neuroinflammation. We established a model of TBI with controlled cortical impacts (CCIs) and a tau hyperphosphorylation model by injecting the virus encoding human P301S tau in mice, and immunofluorescence, western blotting analysis, and behavioral tests were performed to clarify the interaction between phosphorylated tau (p-tau) and HMGB1 levels. We demonstrated that p-tau and HMGB1 were elevated in the spatial memory-related brain regions in mice with TBI and tau-overexpression. Animals with tau-overexpression also had significantly increased nucleotide-binding oligomerization domain-like receptor pyrin domain-containing protein 3 (NLRP3) inflammasome activation, which manifested as increases in apoptosis-associated speck-like protein containing a caspase recruitment domain (ASC), activating caspase-1 and interleukin 1 beta (IL-1β) levels. In addition, NLRP3^–/–^ mice and the HMGB1 inhibitor, glycyrrhizin, were used to explore therapeutic strategies for diseases with p-tau overexpression. Compared with wild-type (WT) mice with tau-overexpression, downregulation of p-tau and HMGB1 was observed in NLRP3^–/–^ mice, indicating that HMGB1 alterations were NLRP3-dependent. Moreover, treatment with glycyrrhizin at a late stage markedly reduced p-tau levels and improved performance in the Y- and T-mazes and the ability of tau-overexpressing mice to build nests, which revealed improvements in spatial memory and advanced hippocampal function. The findings identified that p-tau has a triggering role in the modulation of neuroinflammation and spatial memory in an NLRP3-dependent manner, and suggest that treatment with HMGB1 inhibitors may be a better therapeutic strategy for tauopathies.

## Introduction

The accumulation of phosphorylated tau is a characteristic change in tauopathy and other neurodegenerative diseases. Accumulating research has revealed that tau plays a crucial role in the severity and progression of cognitive impairments (Furman et al., 2017), and that the immunological inhibition of tau has beneficial effects in the therapy of degenerative diseases (Albert et al., 2019; Takeda, 2019; Colin et al., 2020). The hyperphosphorylation of tau is consistently observed in secondary tauopathies, such as chronic traumatic encephalopathy (CTE), traumatic brain injury (TBI), and Alzheimer’s disease (AD; Edwards et al., 2017; Johnson et al., 2017; Rubenstein et al., 2017; VanItallie, 2019), and is regarded as a risk factor for AD and CTE (Washington et al., 2016; Collins-Praino and Corrigan, 2017; Kulbe and Hall, 2017). Recent evidence has shown that behavioral disorders are more closely associated with a high level of p-tau after TBI than with amyloid-beta (Aβ; Chen et al., 2009; Huber et al., 2013; Tweedie et al., 2013; Takahata et al., 2017). These abnormalities related to tau occur as early as several months to years after trauma; thus, hyperphosphorylated tau is considered the “trigger” of AD (Bramlett and Dietrich, 2015; Johnson et al., 2017). In addition, the p-tau level has a dose-dependent effect on the severity and frequency of trauma, suggesting that tau hyperphosphorylation may be a key factor for the early onset or rapid progression of cognitive impairments (Edwards et al., 2017). However, the distribution and spread of p-tau in brain regions varies depending on the type of neurodegenerative disease (Kaufman et al., 2016), and the mechanisms by which abnormal tau leads to neuropathological alterations and cognitive damage have not been fully clarified.

Neuroinflammation is a typical pathological change seen with neurodegenerative diseases, and the relationship between neuroinflammation and characteristic pathological impairments (neurofibrillary tangles caused by tau hyperphosphorylation and senile plaques caused by Aβ) has received much attention from neuroscientists. In particular, the NLRP3 inflammasome, which is involved in both infectious and sterile inflammation, has recently been considered a potential therapeutic target for cognitive impairments such as AD (White et al., 2017; Swanson et al., 2019). A large body of research has shown that NLRP3 inhibition reduces the release of proinflammatory factors and inhibits the activation of microglia in experimental animals in the acute phase after TBI (Kuwar et al., 2019; Yi et al., 2019). A variety of pathogen-associated molecular patterns (PAMPs) and damage-associated molecular patterns (DAMPs) can activate the (Kaufman et al., 2016) NLRP3 inflammasome and cause significant increases in proinflammatory factors such as IL-1β. Recently, it has been found that Aβ is also an activator of NLRP3 (Halle et al., 2008); however, there are limited studies on whether phosphorylated tau (monomer or oligomer) could activate the NLRP3 inflammasome, and the underlying mechanisms remain to be clarified. The activation of the NLRP3 inflammasome leads to neuroinflammation not only by activating caspase-1 to produce interleukin 1 beta (IL-1β) and interleukin-18 (IL-18) but also by releasing high mobility group box-1 (HMGB1) to initiate a cascade of inflammation that can lead to neuroinflammatory responses. Many studies have reported that HMGB1, as an important late-stage inflammatory mediator, is an important therapeutic target for infectious diseases such as sepsis-associated encephalopathy and autoimmune encephalomyelitis (Uzawa et al., 2013) at an early stage. It has been reported that the expression of HMGB1 increases approximately 12 h following TBI, spinal cord injury, and cerebral hemorrhage, and that anti-HMGB1 therapy can reduce brain edema, inhibit neuroinflammation, and restore neurological dysfunction (Okuma et al., 2012; Haruma et al., 2016; Wang et al., 2017; Kobayashi et al., 2018). However, HMGB1 almost returned to the baseline level 2–4 weeks after injury according to published data (Kigerl et al., 2018). It is not clear whether HMGB1 increases in the late period after brain injury. However, HMGB1 is closely related to cognitive impairment in the chronic period after injury. For instance, prolonged increase in HMGB1 is associated with cognitive impairment in intensive care unit survivors (Bruck et al., 2020), suggesting that HMGB1 is an important factor that exacerbates the progression of cognitive impairments. However, the reason for the increase in HMGB1 at a delayed stage and the interaction between the increased p-tau and HMGB1 are not fully understood. Therefore, we tried to identify the effect of hyperphosphorylated tau on the activation of the NLRP3 inflammasome and HMGB1 levels in different brain regions using animals with tau-overexpression and NLRP3 knockout. In addition, we explored the therapeutic strategy of HMGB1 inhibitors to elicit neuroprotective effects in neurodegenerative diseases with tau overexpression. This research paves the way for the multiple-target treatment of neurodegenerative diseases and offers insight into the complexity of p-tau-HMGB1 interactions.

## Materials and Methods

### Animals

Adult male mice (10–11 weeks old), purchased from the Shanghai Laboratory Animal Research Center (Shanghai, China), were housed in a 12-h light/dark cycle facility and allowed free access to food and water. Global NLRP3 knockout mice on a C57BL/6J background were provided by Professor Lei Li from Army Medical University. All the animal experiments were approved by the Laboratory Animal Welfare and Ethics Committee of the Army Medical University (AMUWEC20191822), and complied with the NIH Guide for the Care and Use of Laboratory Animals.

### Traumatic Brain Injury Models

**FIGURE 1:**
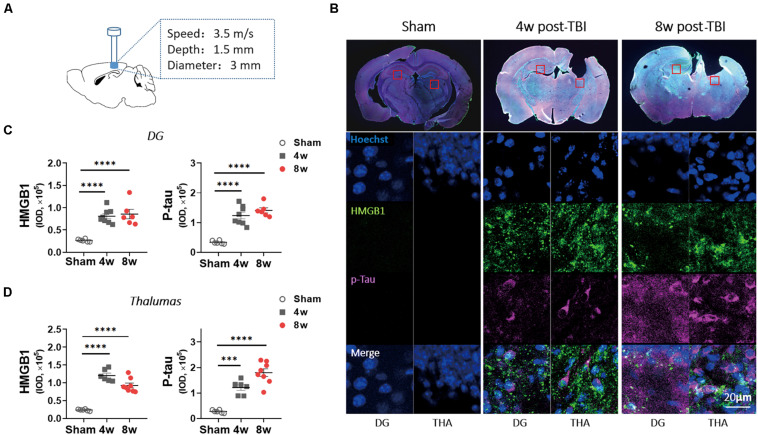
Levels of p-tau and high mobility group box-1 (HMGB1) were increased in wild-type (WT) mice with traumatic brain injury (TBI). **(A)** An experimental mouse model of TBI. **(B)** Representative images showed the increased immunoreactivities for HMGB1 and p-tau (ser404) in ipsilateral thalamus (THA) (close to lesion location) and contralateral dentate gyrus (DG) at 4 and 8 weeks (w) post-TBI. **(C,D)** HMGB1 and p-tau immunoreactivities were quantified with fluorescence intensity in **(C)** DG and **(D)** thalamus 4 and 8 weeks post TBI. One-way analysis of variance (ANOVA) was performed followed by Dunnett’s multiple comparisons test. ^∗∗∗^*p* < 0.001, ^****^p < 0.0001.

The animal model of moderate TBI was established by controlled cortical impact (CCI) according to a published article (Zhao et al., 2017b). Briefly, the mice were anesthetized using 50 mg/kg pentobarbital sodium. TBI was induced using a head trauma contusion device with a pneumatic impactor (TBI-0310, PSI, Hampton, United States) after craniotomy was performed over the left parietal cortex. The impact parameters were a velocity of 3.5 m/s, diameter of 3 mm, and depth of 1.5 mm (Figure 1A). The mice were then placed on an electric blanket until they woke up and then returned to their home cages. In the sham group, only anesthesia and craniotomy were performed in animals.

### Overexpression of Tau P301S

The adeno-associated virus (AAV) encoding human P301S tau under the control of the neuron-specific CaMKII promoter (AAV2/9-CaMKII-hTau^*P*301*S*^-GFP, AAV-tau hereafter) and a control virus (AAV2/9-CamkII-GFP, AAV-GFP hereafter) were created by Hanbio Biotechnology Co. Ltd. (Shanghai, China). The mice were anesthetized, and craniotomy was performed using a.5 mm diameter drill over the left parietal cortex (AP: −2 ML: −1 DV: −1.5). The animals were stereotaxically injected with 1 μl of normal saline (Sham), AAV-tau, or AAV-GFP (viral titers 1.8 × 10^12^ vg/ml) into the hippocampus at a rate of 200 nl/min, and the needle was kept in place for 5 min before retraction. Then, the mice were removed from the stereotaxic frame and returned to their home cages.

### Drugs

Glycyrrhizin, an HMGB1 inhibitor (GZ, Selleck, Shanghai, China), was dissolved in 2% dimethyl sulfoxide (DMSO) + 30% polyethylene glycol (PEG) 300 + 2% Tween 80 + ddH_2_O according to the recommendation of the manufacturer. The mice were injected intraperitoneally with a volume of 100 μl GZ at a dose of 25 mg/kg. The drug was administered once daily for 7 days. Then, the drug was administered once a week for 3 weeks. The total treatment duration was 4 weeks (Figure 7A).

### Behavioral Experiments

**FIGURE 2:**
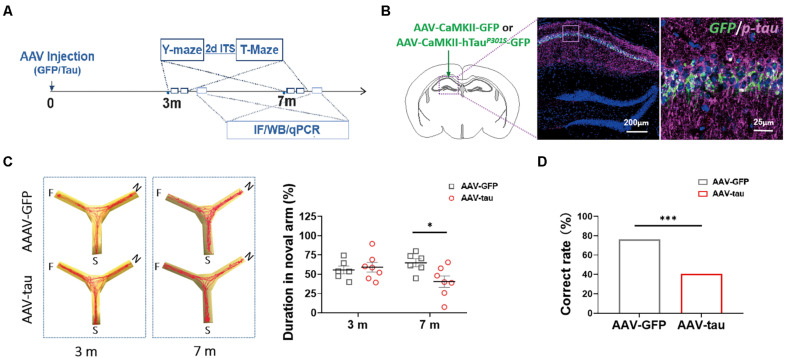
Overexpression of p-tau induced cognitive dysfunction in mice with adeno-associated virus (AAV)-tau injection. **(A)** Experimental paradigm. **(B)** Representative images of the ipsilateral hippocampus taken from mice injected with AAV2/9-CaMKII-hTau^*P*301*S*^-GFP (called AAV-tau) into the hippocampus. The positive staining for phosphorylated tau at ser404 (purple) indicated successful animal model with p-tau overexpression. **(C)** Representative moving tracks (left) and the percentages of time spent in the novel arm (right) of mice that received AAV-tau (*n* = 8) or AAV-GFP (*n* = 6) injections in Y-maze at 3 and 7 months time points. **(D)** Working memory of mice was assessed by the total correct rate of six trials at 3 (*n* = 10) and 7 months (*n* = 7 and 12, AAV-GFP and AAV-tau) post-injection in a T-maze test. Two-way ANOVA was performed followed by Bonferroni’s multiple comparisons test. Pearson chi-square test was also performed, ^∗^*p* < 0.05, ^∗∗∗^*p* < 0.001.

The behavioral tests of mice were performed at the indicated time points (Figures 2A, 7A) and analyzed by the EthoVision XT system (Version 11.5; Noldus, Wageningen, Netherlands). All the tests were conducted by experimenters blinded to the animal groups.

The Y-maze was used to assess short-term spatial memory, as previously described (Webster et al., 2019a). In brief, each trial consisted of a 5-min acquisition phase (novel arms were blocked), an intertrial interval (30 min), and a final 5-min test phase (all arms were open). The total amount of time spent in each arm was recorded, and the percentage of time spent in the novel arm was used to reflect the spatial memory of mice (McAllister et al., 2018). It was calculated as [the time in novel arm/(the time in novel arm + the time in familiar arm)] × 100.

We used the T-maze to assess the working memory of the animals. There were two phases in the current paradigm: a sample phase and a choice phase with a 1-min interval (Deacon and Rawlins, 2006). The animals were placed in the start area, allowed to choose a goal arm, and confined in the chosen arm for 1 min. Then, the animals were returned to the start arm to undergo another choice, and a different choice against the former was recorded as correct. We performed the test twice a day for three consecutive days. The total percentage of correct responses was calculated to measure the performances in this behavioral testing.

The capacity of nest building reflected advanced hippocampus cognition. Briefly, the mice were housed individually, and a piece of cotton material was placed in each cage overnight for them to build nests. Then, the nests were assessed the next morning by weighing untorn cotton pieces (Deacon, 2006). A higher percentage of untorn material indicated nest building performances that were worse.

### Immunofluorescence Staining

The mice were anesthetized with 1.5% sodium pentobarbital and perfused transcardially with phosphate-buffered saline (PBS), followed by 4% paraformaldehyde (PFA). Their brains were dissected and fixed in 4% PFA for 24 h, and coronal cryosections (30 μm thick) were obtained from the experimental animals. Anti-tau Ser404 (ab92676) and anti-HMGB1 (ab18256) antibodies were purchased from Abcam (Cambridge, United Kingdom), and anti-NLRP3 (AG-20B-0014-C100), anti-ASC (AG-25B-0006-C100), and anti-caspase-1 (P20) (AG-20B-0042-C100) antibodies were purchased from AdipoGen Life Sciences (San Diego, CA, United States) (detailed information in Supplementary Table 1). Fluorescence images were acquired using a laser scanning confocal fluorescence microscope (TCS SP8; Leica, Wetzlar, Germany). The fluorescence images were converted to 8-bit images, and then the stained area was quantified using the ImageJ software. Detection of the background staining was eliminated by setting the same threshold to all the images.

### Western Blotting

The mouse brains were rapidly dissected on ice, and the hippocampal and prefrontal cortices were removed for western blotting. The tissues were homogenized using T-PER Tissue Protein Extraction Reagent (Thermo Fisher Scientific, Waltham, MA, United States) plus Pierce Protease and Phosphatase Inhibitor Mini Tablets (Thermo Fisher Scientific, Waltham, MA, United States). The proteins were separated by 10% sodium dodecyl sulfate-polyacrylamide gel electrophoresis (SDS-PAGE) electrophoresis, transferred onto polyvinylidene difluoride (PVDF) membranes, and incubated overnight in primary antibodies. Except for the anti-tau 5 (ab80579; Abcam, Cambridge, United Kingdom) and anti-glyceraldehyde 3-phosphate dehydrogenase (GAPDH) (Bioworld, St. Louis, Park, MN, United States) antibodies, the rest were the same as those in immunofluorescence experiments. The bands were analyzed using the ImageJ software.

### Quantitative Reverse Transcription Polymerase Chain Reaction

Total RNA was extracted using an Eastep^®^ Super Total RNA Extraction Kit (Promega, Madison, WI, United States) and then reverse transcribed to cDNA (GoScript^TM^ Reverse Transcription System; Promega, Madison, WI, United States) according to the instructions of the manufacturer. Quantitative polymerase chain reaction (qPCR) was performed in a Stratagene Mx3000P system (Stratagene, La Jolla, CA, United States). The primer sequences were as follows (detailed information in Supplementary Table 2): IL-1β: acggaccccaaaagatgaag and ttctccacagccacaatgag; IL-18: gcctcaaaccttccaaatcac and gttgtctgattccaggtctcc; tumor necrosis factor α (TNF-α): cttctgtctactgaacttcggg and caggcttgtcactcgaattttg; GAPDH: ctttgtcaagctcatttcctgg and tcttgctcagtgtccttgc. The results presented are normalized to sham.

### Statistical Analysis

Two-tailed unpaired Student’s *t*-test was performed to compare the two groups. One- or two-way ANOVA was performed for experiments containing more than two groups, followed by Bonferroni’s multiple comparisons test. Data are shown as mean ± standard error of mean (SEM). Pearson chi-square test was performed for rate comparison. Differences were considered to be statistically significant at *p* < 0.05. All the data were analyzed using the Prism 5.0 software.

## Results

### Traumatic Brain Injury Causes Persistent Increases in HMGB1 and p-Tau Levels

To confirm the role of HMGB1 in the delayed period (4–8 weeks post injury), a period during which cognitive disorders appeared after TBI, immunofluorescence examination was performed in the mice with moderate TBI. Although the ipsilateral hippocampus was lacking, a significant increase in HMGB1 immunoactivity was observed at 4 weeks post injury in the contralateral dentate gyrus (DG) and ipsilateral thalamus compared with the sham group, and this change existed for 8 weeks after the brain injury (Figures 1B–D). As the previous study found that p-tau (Ser404) increased obviously after TBI in mice, we investigated p-tau and found that it increased significantly in the above two brain regions at 4 and 8 weeks post injury (Figures 1B–D). Similar trends in p-tau and HMGB1 changes led to speculation that p-tau may cause elevation of HMGB1.

### Overexpression of Phosphorylated Tau Induces Late-Onset Cognitive Dysfunction

To understand whether hyperphosphorylated tau following TBI alters HMGB1 levels independently, we established a tau-overexpressing (mostly phosphorylated) model by AAV-tau injection. The immunofluorescence staining revealed obvious elevations in endogenous (only p-tau positive) and exogenous p-tau proteins (both p-tau and GFP positive) in the left hippocampus ipsilateral to the injection site 3 m after injection (Figure 2B).

In the Y-maze, no significant differences were detected between the tau-overexpressing group and the GFP control at 3 m after injection. At 7 m, the tau-overexpressing mice displayed significant spatial cognitive impairment, with reduced percentage of exploration time spent with the novel arm compared with the control (Figure 2C). As with the T-maze test, the tau-overexpressing animals showed worse performance at 7 m, suggesting that working memory deficits occurred at a relatively late period post injection (Figure 2D).

### Overexpression of p-Tau in the Hippocampus Leads to Elevations in HMGB1

**FIGURE 3:**
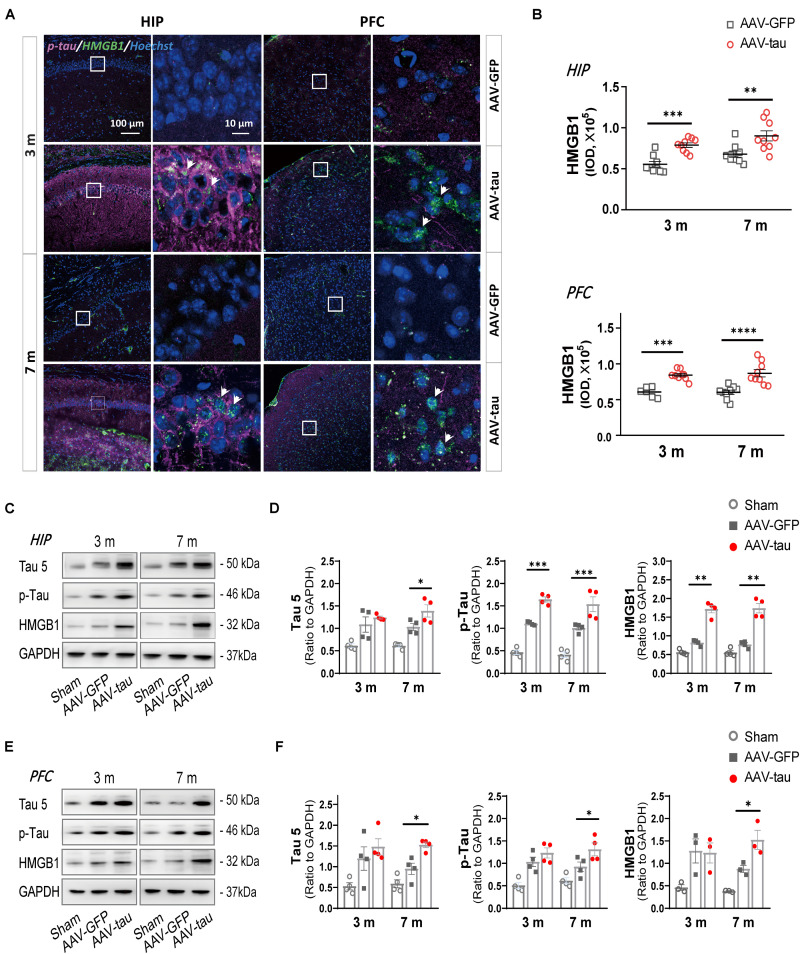
Overexpression of p-tau led to increases in HMGB1 and tau expressions. **(A)** Representative images of HMGB1 (green) and p-tau (ser404) (purple) in the ipsilateral hippocampus (HIP) and prefrontal cortex (PFC) taken from mice injected with AAV-tau or AAV-GFP 3 and 7 months after injection. Right columns of HIP and PFC are higher magnification sections of the dashed squares in the left columns, and white arrows indicate positive staining for p-tau or HMGB1. **(B)** Quantification of HMGB1 with fluorescence intensity (nine regions of interest from three mice for every group) showed significant increases in HMGB1 levels in the HIP (top) and PFC (bottom). **(C–F)** Representative western blots of tau 5 (total tau), p-tau (ser404), and HMGB1 in **(C)** the ipsilateral hippocampus and **(E)** PFC. Quantifications by the integrated optical density showed increased immunoreactivities for tau 5 (left), p-tau (middle), and HMGB1 (right) in the **(D)** HIP and **(F)** PFC of mice with AAV-tau or AAV-GFP 3 and 7 months after virus injection, and data were normalized to glyceraldehyde 3-phosphate dehydrogenase (GAPDH), *n* = 3–4 per group. Two-way ANOVA was performed followed by Bonferroni’s multiple comparisons test for behavioral analysis, ^∗^*p* < 0.05, ^∗∗^*p* < 0.01, ^∗∗∗^*p* < 0.001, ^****^p < 0.0001.

To determine whether the increased p-tau prompted the alteration in HMGB1 expression, this experiment detected HMGB1 levels in spatial memory-related regions, the hippocampus and prefrontal cortex (PFC). Compared with the GFP control, HMGB1 levels were elevated in the ipsilateral hippocampal CA1 region and PFC at both 3 and 7 months post injection in the AAV-tau groups (Figures 3A–F). Moreover, the expression of total tau (tau 5) and p-tau (Ser404) was significantly increased in the hippocampus (Figures 3C,D) and PFC (Figures 3E,F) at a relatively late stage of 7 months. Of note, the levels of the above proteins increased over time, and the increases were more pronounced at 7 months post injection when cognitive impairments were present, implying the underlying relationship between elevated HMGB1 induced by p-tau overexpression and spatial memory disorders.

### p-Tau Overexpression Causes Activation of the NLRP3 Inflammasome

Studies have shown that the activation of inflammasomes is related to the release of HMGB1 (Yu et al., 2019). Therefore, the most extensively explored NLRP3 inflammasome, which is well known to affect cognition, was detected by immunofluorescence staining and western blot. We detected components of the NLRP3 inflammasome, such as NLRP3, ASC, caspase-1, and its active isomer P20.

**FIGURE 4:**
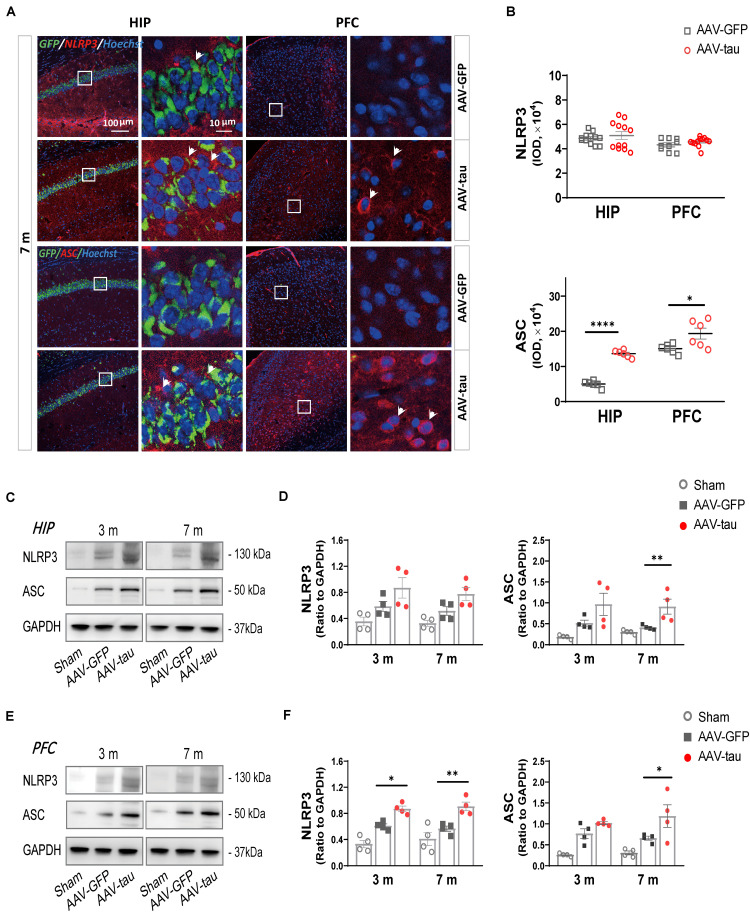
Overexpression of p-tau caused elevations in ASC expression in the hippocampus and PFC. **(A)** Representative images of nucleotide-binding oligomerization domain (NOD)-like receptor pyrin domain-containing protein 3 (NLRP3) (red) and ASC (red) in the ipsilateral HIP and PFC taken from mice injected with AAV-tau or AAV-GFP 3 and 7 months after injection. White arrows indicate positive staining for NLRP3 or ASC. **(B)** Quantifications of NLRP3 (top) and ASC (bottom) immunoactivities shown in the previous panel (6–12 ROIs from three mice for every group). **(C–F)** Representative western blots of NLRP3 and ASC in the **(C)** ipsilateral HIP and **(E)** PFC, and quantifications of the immunoreactivities for NLRP3 (left) and ASC (right) in the **(D)** HIP and **(F)** PFC. Data were normalized to GAPDH, *n* = 4 per group. **(D,F)** Two-way ANOVA and **(B)** unpaired *t*-test were performed, ^∗^*p* < 0.05, ^∗∗^*p* < 0.01, ^****^p < 0.0001.

There were no significant differences in NLRP3 immunoactivity between the AAV-tau and AAV-GFP groups in the hippocampus and prefrontal cortex at 7 m. In contrast to NLRP3, mice with AAV-tau displayed considerable increases in ASCs in the hippocampus and PFC by immunoactivity and western blot analyses (Figures 4A,B). In addition, NLRP3 expression was significantly increased in the PFC at 3 and 7 m but not in the hippocampus (Figures 4C–F).

**FIGURE 5:**
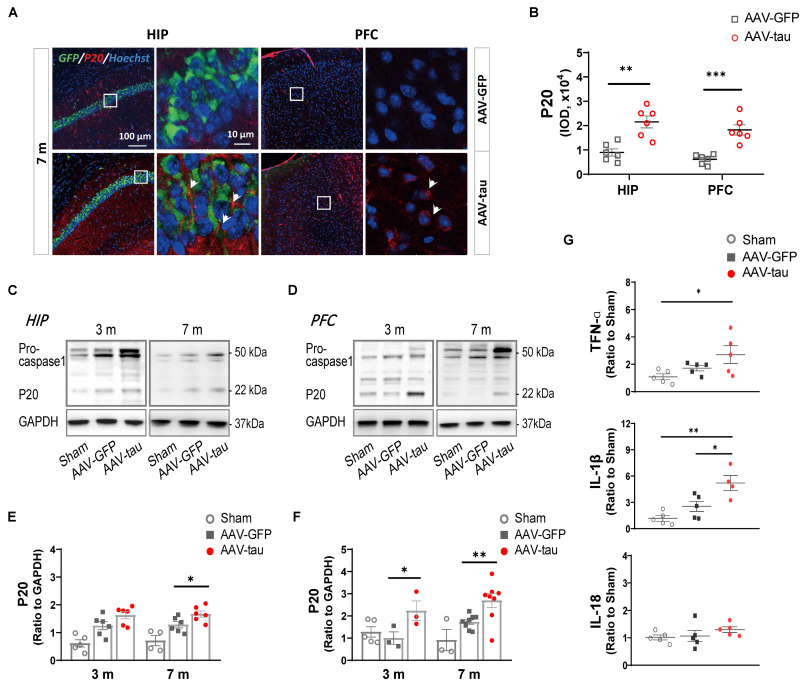
Overexpression of p-tau caused the activation of NLRP3 inflammasome in the hippocampus and PFC. **(A)** Representative images and **(B)** quantifications of P20 immunostainings (six ROIs from three mice for every group) in the HIP and PFC in mice injected with AAV-tau or AAV-GFP 7 months after injection. White arrows indicate positive staining for P20. **(C,D)** Representative western blots of P20 and their quantifications by the integrated optical density in the **(E)** HIP and **(F)** PFC from mice receiving AAV-tau or AAV-GFP control 3 and 7 months after injection. Data were normalized to GAPDH, *n* = 3–8 per group. **(G)** Messenger RNA (mRNA) expression of tumor necrosis factor α (TNF-α) (top), interleukin 1 beta (IL-1β) (middle), and interleukin-18 (IL-18) (bottom) in the HIP of mice injected with AAV-tau or AAV-GFP 7 months after injection, *n* = 4–5 per group. **(B)** Unpaired *t*-test (b), **(G)** one-way ANOVA, and **(E,F)** two-way ANOVA were performed, ^∗^*p* < 0.05, ^∗∗^*p* < 0.01, ^∗∗∗^*p* < 0.001.

To evaluate the activation of the NLRP3 inflammasome, P20 (cleaved caspase-1) and proinflammatory factors were detected. The results revealed that P20 levels were dramatically elevated in the hippocampus and PFC at 7 m (Figures 5A–F). The mice with tau overexpression displayed a significant increase in IL-1β compared with the AAV-GFP control, but no significant change was observed in the messenger RNA (mRNA) level of IL-18 at 7 m post injection (Figure 5G).

### NLRP3 Knockout Reduces Elevations in HMGB1 and p-Tau Levels

**FIGURE 6:**
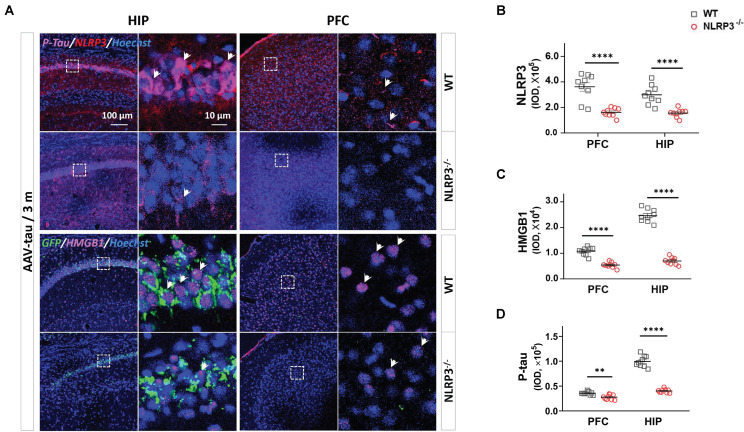
NLRP3^– /–^ mice displayed the lower levels of p-tau and HMGB1 after AAV-tau injection. **(A)** Representative images and quantifications of **(B)** NLRP3, **(C)** HMGB1, and **(D)** p-tau (ser404) immunostainings in the HIP and PFC (nine ROIs from three mice for every group) of mice injected with AAV-tau or AAV-GFP 3 months after injection. White arrows indicate positive staining for p-tau or HMGB1. Unpaired *t*-test was performed for immunohistochemical analysis, ^∗∗^*p* < 0.01, ^****^p < 0.0001. WT, wild-type.

We next confirmed whether the increased HMGB1 levels in the hippocampus and PFC after tau overexpression were caused by the activation of the NLRP3 inflammasome in the NLRP3^–/–^ mice. The results showed that the NLRP3 staining was almost invisible in the hippocampus and PFC after NLRP3 gene knockout, indicating success of the animal model (Figures 6A,B), and the expression of HMGB1 in mice receiving AAV-tau virus was also significantly lower than that in their wild-type (WT) littermates in these two brain regions 3 m post injection (Figures 6A,C). Importantly, p-tau immunoactivities were significantly decreased in both the hippocampus and PFC of the NLRP3^–/–^ mice relative to the WT mice (Figures 6A,D).

### Glycyrrhizin Treatment Prevents Pathological Alterations of Tau and Alleviates Neurobehavioral Deficits in Tau-Overexpressing Mice

**FIGURE 7:**
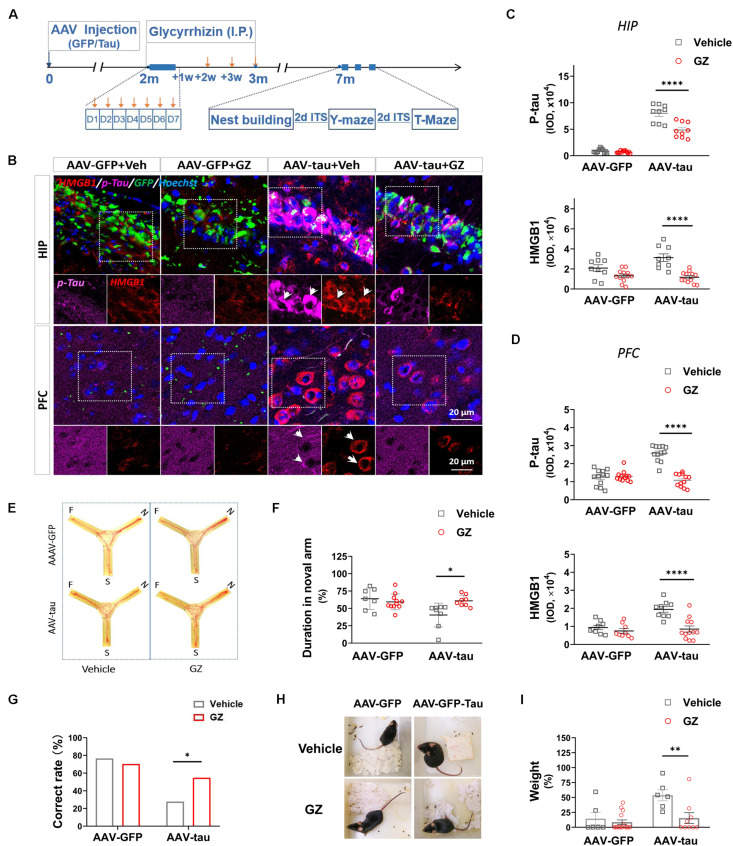
High mobility group box-1 inhibitor alleviated tau pathology and improved spatial cognition in mice with p-tau overexpression. **(A)** Experimental procedure and drug administration schemes. Glycyrrhizin (GZ, 25 mg/kg i.p.), a pharmacological inhibitor of HMGB1, or vehicle was administered 2 months after virus injection, and orange arrows indicate the injection time. **(B)** Representative images of p-tau (ser404) and HMGB1 in the HIP and PFC from mice receiving AAV-tau or AAV-GFP 7 months after injection. White arrows indicate positive staining for p-tau or HMGB1. **(C,D)** Quantifications of p-tau (top) and HMGB1 (bottom) immunostainings (9∼12 ROIs from three mice for every group) in the **(C)** HIP and **(D)** PFC shown in panel **(B)**. **(E)** Representative moving tracks and the percentages of duration in **(F)** novel arm in GZ or vehicle-treated mice that received AAV-tau (*n* = 8) or AAV-GFP (*n* = 7 for vehicle and *n* = 11 for GZ) injections in the Y-maze test. **(G)** GZ improved the performance of p-tau-overexpressed mice in the T-maze test. **(H)** Representative nest pictures and **(I)** weight analysis of the cotton pads used for nest building showed GZ decreased the weight percentage of untorn cotton in mice that received AAV-tau injections. **(G)** Two-way ANOVA and **(C,D,F,I)** Pearson chi-square test were performed,^∗^*p* < 0.05,^∗∗^*p* < 0.01, ^****^p < 0.0001.

We next examined the effect of the HMGB1 inhibitor GZ on cognitive disorders in the delayed period following virus injection. The GZ treatment was initiated 2 months (m) post injection and maintained for 1 month. This therapeutic strategy reduced the immunofluorescence staining for HMGB1 compared with vehicle-treated controls in the hippocampus and PFC of tau-overexpressing mice 7 m post injection. Surprisingly, the treatment also decreased the p-tau staining at the above regions at 7 m (Figures 7B–D), implying that the inhibition of HMGB1 was beneficial to rescue the elevated p-tau in the hippocampus and PFC. The next behavioral tests demonstrated that the GZ treatment significantly improved the spatial short-term memory of the animals in the AAV-tau group compared with the vehicle-treated mice by the Y-maze task (Figures 7E,F), and that the performance of the HMGB1-administered mice in the T-maze task was also better than that of the vehicle-treated ones, suggesting restored hippocampal-spatial working memory (Figure 7G). In addition, nest building activity, considered to be a manifestation of advanced hippocampal cognition, was recovered after the GZ treatment (Figures 7H,I).

## Discussion

### Elevated p-Tau and HMGB1 Levels in Spatial Memory-Related Brain Regions Post-TBI Associated With Late-Onset Cognitive Deficits

High mobility group box-1, as a late inflammatory factor and immunoregulatory molecule, has been reported to be elevated during the acute and subacute phases (3–12 h) of brain injury and to gradually return to normal levels up to 4 weeks post injury (Kigerl et al., 2018; Webster et al., 2019b). In this study, it was found that HMGB1 increased 4 and 8 weeks after TBI, which was consistent with the temporal and spatial distribution of increased p-tau at Ser404, suggesting that increased HMGB1 in a delayed period was closely related to tau hyperphosphorylation. Mounting evidence has revealed that TBI induces increases in p-tau levels *via* a variety of factors, such as detachment of p-tau from microtubules induced by mechanical forces, dissociation caused by hyperphosphorylation (Collins-Praino and Corrigan, 2017), and neuroinflammation resulting from axonal injury (Smith et al., 2003). An increasing number of studies have highlighted how elevated p-tau results in neuropathological changes and cognitive impairments and whether endogenous immune-related molecules are involved in the detrimental roles of p-tau in cognition. Although the accumulating literature has reported that HMGB1 is closely related to cognitive dysfunction caused by a variety of reasons, such as neurodegenerative diseases (Hei et al., 2018; Okuma et al., 2019), surgery (Kong et al., 2017), and sepsis (Chavan et al., 2012), the association between p-tau and HMGB1 in a delayed period post injury remains undefined. Here, we found that HMGB1 and p-tau (Ser404) increased simultaneously during the period when spatial memory impairment occurred, suggesting that the overexpression of p-tau may be mainly responsible for an earlier onset of spatial cognitive dysfunction in patients with TBI rather than in elderly patients with AD through the promotion HMGB1 levels in spatial memory-related brain regions.

### p-Tau Is an Independent Factor for Increased HMGB1 and Decreased Cognition

It has been well demonstrated that sterile and infectious threats can cause an increase in HMGB1 levels in the central nervous system, which is associated with the activation of microglia and astrocytes, and the release of proinflammatory cytokines, such as TNF-α and IL-1β. Considering the influence of various stimuli on HMGB1 after TBI, this study established an animal model with high expression of p-tau by injecting AAV-tau into the hippocampus of mice to clarify the influence of p-tau on HMGB1. Behavioral tests indicated that tau-overexpressing animals presented spatial cognitive dysfunction 7 m post injection, and most importantly, alterations in total tau, p-tau, and HMGB1 appeared in the spatial memory-related brain regions, hippocampus and PFC at the same time point. Of note, the increase in the phosphorylation of tau at Ser404 was obvious after TBI, which has been demonstrated in the previously published article (Zhao et al., 2017a). Tau phosphorylation at Ser404 occurs more frequently after TBI than in neurodegenerative diseases such as AD, in which the phosphorylation sites at Thr205, Thr181, Ser262, and Tyr396 are much more common (Katsumoto et al., 2019; Barthelemy et al., 2020). Several pieces of evidence have demonstrated that Ser404 phosphorylation is involved in the aggregation and mislocalization of tau; thus, we speculated that Ser404 phosphorylation may be facilitated by the disassociation of tau from microtubules in injured axons following TBI. Importantly, we found that endogenous tau phosphorylation at Ser404 was also markedly increased in animals that received P301S mutant human tau. These results suggest that p-tau may act as an independent factor causing neuropathologic changes.

High mobility group box-1, regarded as a late proinflammatory cytokine, triggers inflammatory response through several receptors, such as toll-like receptor-4 (TLR4), TLR2, and the receptor for advanced glycation end product (RAGE). It is also a non-histone chromatin DNA-binding protein that, therefore, regulates transcription factor recruitment. HMGB1 is mainly expressed in the nucleus of hippocampal neurons (Faraco et al., 2007). In this study, HMGB1 was obviously increased in the hippocampus and PFC, regions tightly associated with spatial cognition, suggesting that HMGB1 may act as a detrimental downstream factor of p-tau in the cognitive impairment caused by tau overexpression. We, therefore, speculated that p-tau might be an initiator that triggers a series of neuropathological alterations in tauopathies.

### Increases in HMGB1 Induced by Tau-Overexpression Were Dependent on the Activation of NLRP3 Inflammasome

Recent evidence indicates that the activation of the nucleotide-binding oligomerization domain-like receptor pyrin domain-containing protein 3 inflammasome is one of the most important factors for the release of high mobility group box-1 and is widely involved in cognitive dysfunction in patients with AD and animal models (Singhal et al., 2014; Pennisi et al., 2017; Thawkar and Kaur, 2019). The NLRP3 inflammasome is composed of three components: a cytosolic pattern recognition receptor (NLRP3), an adaptor protein (ASC), and an effector component (caspase-1). In this study, the increases in ASC specks showed that more constituent proteins were assembled into the complete NLRP3 inflammasome, and the formation of P20 and the elevation of the downstream proinflammatory factor IL-1β suggested the activation of the NLRP3 inflammasome. Many studies have reported that the NLRP3 inflammasome is involved in HMGB1 elevation in sepsis (Lamkanfi et al., 2010), inflammatory kidney disease (Zhang et al., 2020), acute lung injury (Hou et al., 2018), and other infectious diseases (Willingham et al., 2009; Yu et al., 2019), and most studies have focused on immune cells such as macrophages and microglia.

In the central nervous system, Aβ activates the nucleotide-binding oligomerization domain-like receptor pyrin domain-containing protein 3 inflammasome in microglia and further aggravates the phosphorylation of tau (Ising et al., 2019; Lempriere, 2020). However, Aβ was only found in 30% of postmortem acute TBI cases and 40–45% of CTE cases (Edwards et al., 2017). A recent study revealed an exacerbating role of the ASC-NLRP3 axis in seeded tau pathology in primary microglia and in tau mice (Stancu et al., 2019), but it is still unclear whether the overexpression of p-tau in the cytoplasm could cause the alteration of HMGB1 and the activation of the NLRP3 inflammasome before they spread through the extracellular space or innervated nerve fibers to other brain regions. In this study, the alteration of NLRP3 levels was not obvious, but the NLRP3 inflammasome was activated 7 m post injection, and cognitive impairment appeared, indicating that it may be a major contributor to the elevation in HMGB1 and abnormal cognition resulting from tau overexpression. By the injection of AAV-tau into the NLRP3^–/–^ mice, HMGB1 levels were significantly decreased in the hippocampus and PFC, implying that tau overexpression induced increases in HMGB1 levels, at least partially through the activation of NLRP3. Interestingly, the NLRP3 knockout blocked not only the upregulation of HMGB1 but also the hyperphosphorylation of tau, which suggested that increased HMGB1 could in turn affect tau phosphorylation. HMGB1 promotes tau pathology through the activation of major tau kinases, such as glycogen synthase kinase-3β (GSK-3β), by binding to its receptors (Li et al., 2012; Chen et al., 2014). Therefore, the interaction between p-tau and HMGB1 leads to a vicious cycle in which p-tau causes an increase in HMGB1, and elevated HMGB1 in turn enhances the phosphorylation of tau and ultimately impairs spatial cognition.

### HMGB1 Inhibitor Reduced p-Tau Levels and Alleviated Cognitive Dysfunction

Given the significant increase in HMGB1 caused by p-tau and possible mutual enhancement between these two proteins, we explored the therapeutic effects and optimal administration (timing of treatment initiation and duration) of the HMGB1 inhibitor on behavioral outcomes and neuropathological consequences. HMGB1 levels continued to increase at a late stage, which provided a longer time window for treatment than with most of the inflammatory factors, such as TNF-α and IL-1β. In addition, GZ was revealed to pass through the blood-brain barrier and exert neuroprotective roles by reducing extracellular and intracellular HMGB1 levels. Here, we found that GZ reduced p-tau and HMGB1 levels simultaneously in spatial memory-associated regions, the hippocampus and PFC, and alleviated cognitive dysfunction, suggesting a close association between tau and HMGB1 in the development of cognitive impairment.

Several lines of evidence have indicated that the HMGB1 inhibitor GZ has a good therapeutic effect at doses of 10–50 mg/kg in the early stages after TBI (Sun et al., 2018; Thakur et al., 2020). Based on these data, we utilized a dose of 25 mg/kg in the mice that received AAV-tau and a longer period of treatment (once daily for 7 days followed by once a week for 3 weeks) in this study. We found that the cognitive deficits were alleviated, manifesting as improved abilities of nest building, prolonged times with the novel arm, and higher performances in the Y-maze by this treatment regime. These results suggest that the time window of GZ may not be limited to the acute or subacute phases, and that the delayed administration of GZ can still play a good therapeutic role under tau-overexpression conditions. Moreover, the reduced HMGB1 was accompanied by decreases in p-tau levels after the treatment, which may account for the beneficial effects of the GZ treatment on cognitive impairments. It is plausible to believe that GZ plays a multi-target role in the treatment of tauopathies.

The elevation in HMGB1 levels was observed not only in the animal model of tau overexpression but also in other neurodegenerative diseases. For example, recent studies found that Aβ-induced increases in HMGB1 levels in experimental animals or cultured microglial cells (Nan et al., 2019), but therapy targeting Aβ was not always effective in cognitive disorders. Several studies revealed that Aβ caused the phosphorylation of tau after severe TBI (Tran et al., 2011; Bloom, 2014); therefore, we postulated that the alteration of HMGB1 and the enhancement of the interaction between HMGB1 and p-tau caused by increased p-tau might be key factors responsible for the ineffectiveness of the Aβ antibody. Moreover, Tran et al. found that the elevation in p-tau levels did not recover after the administration of an Aβ antibody (Tran et al., 2011), which supports the hypothesis of the authors. Of note, there might be crosstalk between neurons and glial cells, which affects the interactions between HMGB1 and p-tau, which is worthy of consideration in future studies.

### Study Limitations and Perspectives

Accelerated neurodegeneration after TBI occurs with a complex interplay of pathological mechanisms. The lack of experimental data on neuron- or microglial-specific NLRP3 knockout makes it difficult to draw definitive conclusions regarding the mechanisms by which p-tau overexpression leads to cognitive impairment, which is worthy of further research in the future. In addition, there is some evidence that the biological and behavioral responses to TBI may differ between sexes, and male mice have more intense activation of the NLRP3 inflammasome (Kodama et al., 2020; O’Brien et al., 2020). Therefore, further experimental confirmation in females is needed to identify the effectiveness of HMGB1 antagonists.

## Conclusion

To the best knowledge of the authors, this study is the first to reveal that tau overexpression causes a long-lasting increase in HMGB1 levels through activation of the nucleotide-binding oligomerization domain-like receptor pyrin domain-containing protein 3 inflammasome. In this study, both the HMGB1 inhibitor and the NLRP3 knockout markedly alleviated cognitive deficits. This study provides experimental evidence for the delayed treatment of diseases mainly manifested as hyperphosphorylation of tau, such as CTE, TBI, and frontotemporal dementia, which may better control the progress of the diseases and offer new insight into cognitive impairment.

## Data Availability Statement

The original contributions presented in the study are included in the article/Supplementary Material, further inquiries can be directed to the corresponding author.

## Ethics Statement

The animal study was reviewed and approved by the Laboratory Animal Welfare and Ethics Committee of the Army Medical University.

## Author Contributions

YZ and S-WT conceived and designed the research. Z-ZH and F-BS performed the experiments. PL and Y-LN interpreted the results of experiments. S-YY, Z-AZ, and HD analyzed the data. NY, YP, and XC prepared the figures. Y-GZ drafted, edited, and revised the manuscript. All the authors read and approved the final version of the manuscript.

## Conflict of Interest

The authors declare that the research was conducted in the absence of any commercial or financial relationships that could be construed as a potential conflict of interest.

## Publisher’s Note

All claims expressed in this article are solely those of the authors and do not necessarily represent those of their affiliated organizations, or those of the publisher, the editors and the reviewers. Any product that may be evaluated in this article, or claim that may be made by its manufacturer, is not guaranteed or endorsed by the publisher.
