# Supplementary material for: NLRP3 Inflammasome-Dependent Increases in High Mobility Group Box 1 Involved in the Cognitive Dysfunction Caused by Tau-Overexpression
Source: Front Aging Neurosci. 2021 Sep 3;13:721474. doi: 10.3389/fnagi.2021.721474 (PMC8446370; doi:10.3389/fnagi.2021.721474)

**Supplementary Fig.1**


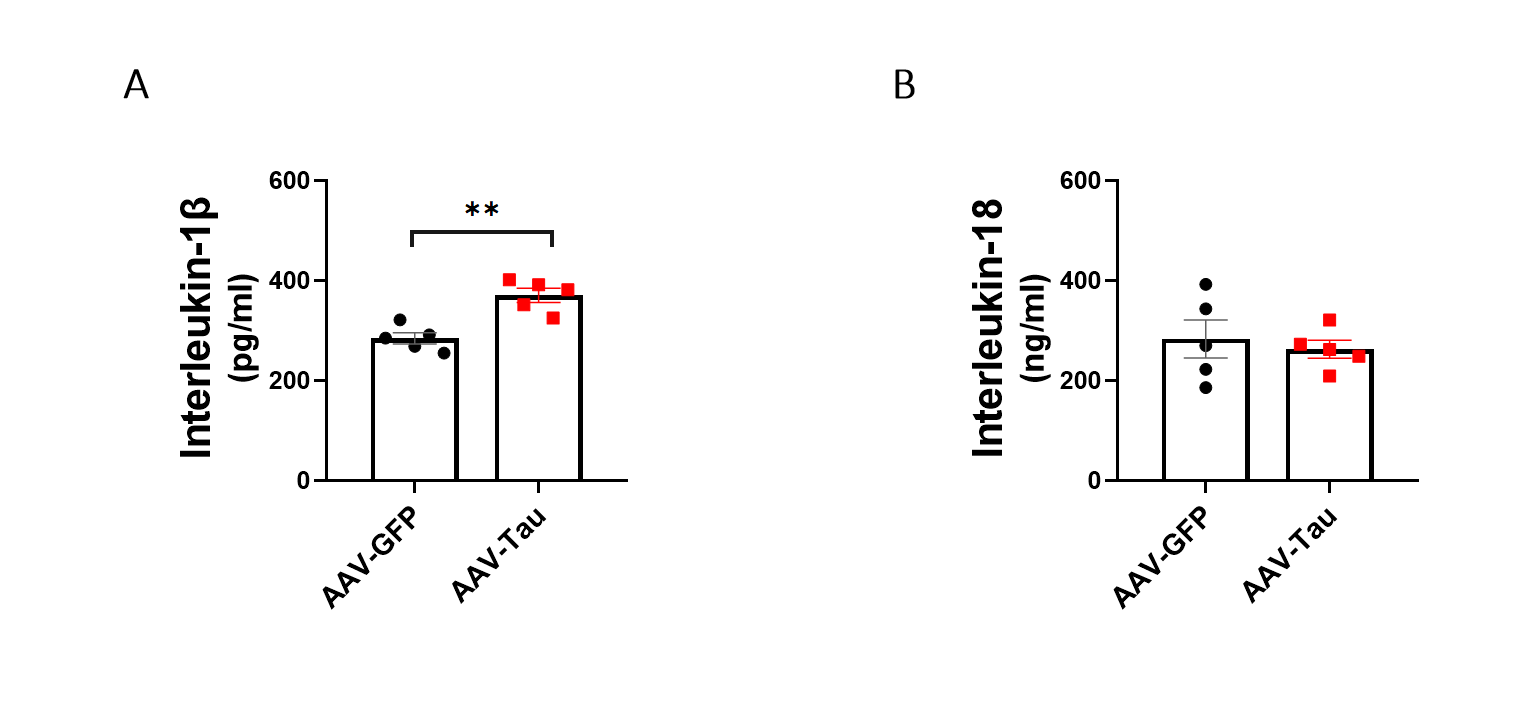


**Supplementary Fig. 1** The changes of inflammatory factors in the hippocampus of AAV-GFG/AAV-Tau mice. (A) IL-1β and (B) IL-18 levels of hippocampal tissue lysate were detected using ELISA kits. (IL-1β and IL-18 Mouse ELISA kit #A201B81053 and #A21880733, MULTI SCIENCES BIOTECH, Hangzhou, CHN). ***p*=0.0014.

| **Supplementary Tab. 1. Primary antibodies used in this study** | | | | | | |
| --- | --- | --- | --- | --- | --- | --- |
| Name | Clonality | Origin | Vendor | Catalog number | Dilution | |
|  |  |  |  |  | WB | IF |
| Tau (TAU-5) | Mono | Mouse | Invitrogen | AHB0042 | 1:1000 |  |
| P-tau Ser404 | Mono | Rabbit | Abcam | ab92676 | 1:1000 | 1:200 |
| NLRP3 | Mono | Mouse | AdipoGen | AG-20B-0014-C100 | 1:1000 | 1:200 |
| ASC | Mono | Rabbit | AdipoGen | AG-25B-0006-C100 | 1:1000 | 1:200 |
| Caspase1 (P20) | Mono | Mouse | AdipoGen | AG-20B-0042-C100 | 1:1000 | 1:200 |
| HMGB1 | Mono | Mouse | Invitrogen | MA5-17278 | 1:1000 | 1:200 |
| GAPDH | Mono | Mouse | Bioworld | MB001H | 1:5000 |  |

| **Supplementary Tab. 2. Primers used in this study** | | | | |
| --- | --- | --- | --- | --- |
| Gene Name | | Accession No. | | Sequences |
| IL-1β | NM_008361 | | Forward: acggaccccaaaagatgaag and Reverse: ttctccacagccacaatgag | |
| IL-18 | NM_008360 | | Forward: gcctcaaaccttccaaatcac and Reverse: gttgtctgattccaggtctcc | |
| TNF-α | NM_013693 | | Forward: cttctgtctactgaacttcggg and Reverse: caggcttgtcactcgaattttg | |
| GAPDH | NM_008084 | | Forward: ctttgtcaagctcatttcctgg and Reverse: tcttgctcagtgtccttgc | |

**Full blot images of Fig. 3C, E**

**
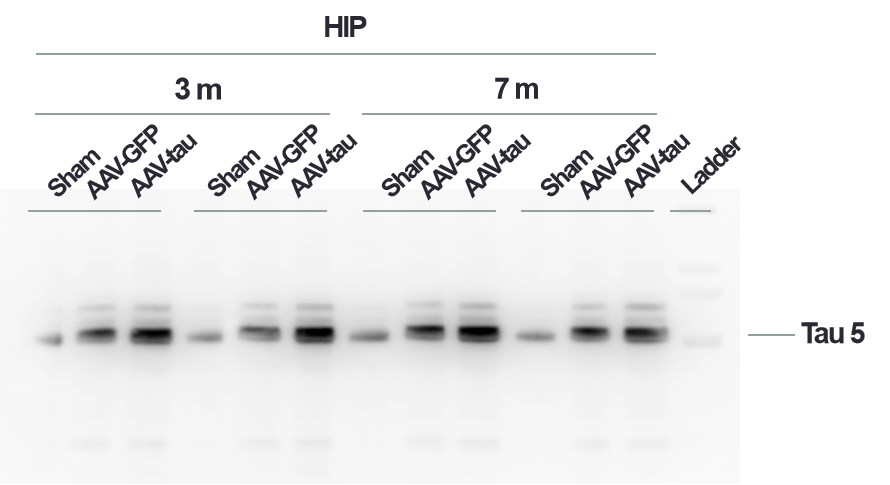

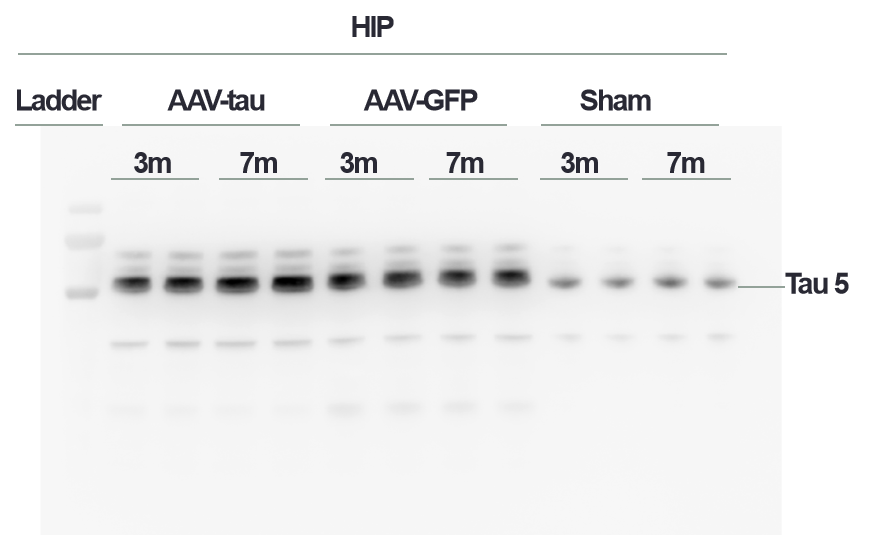
**

**
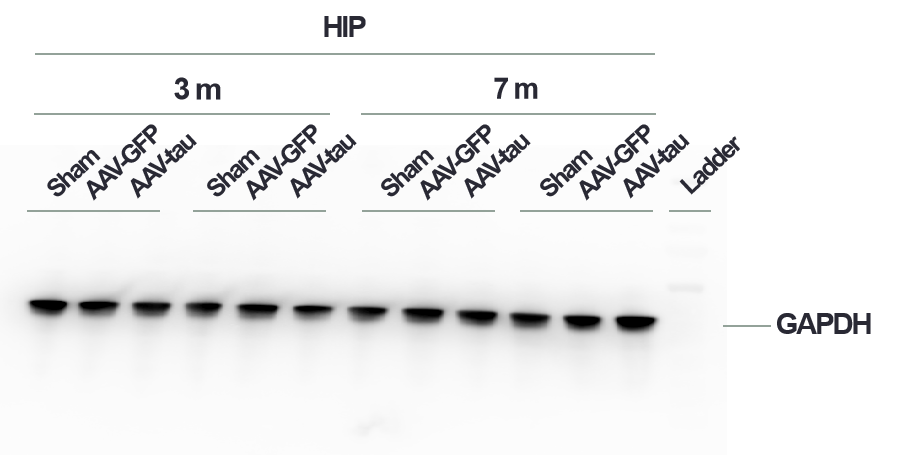

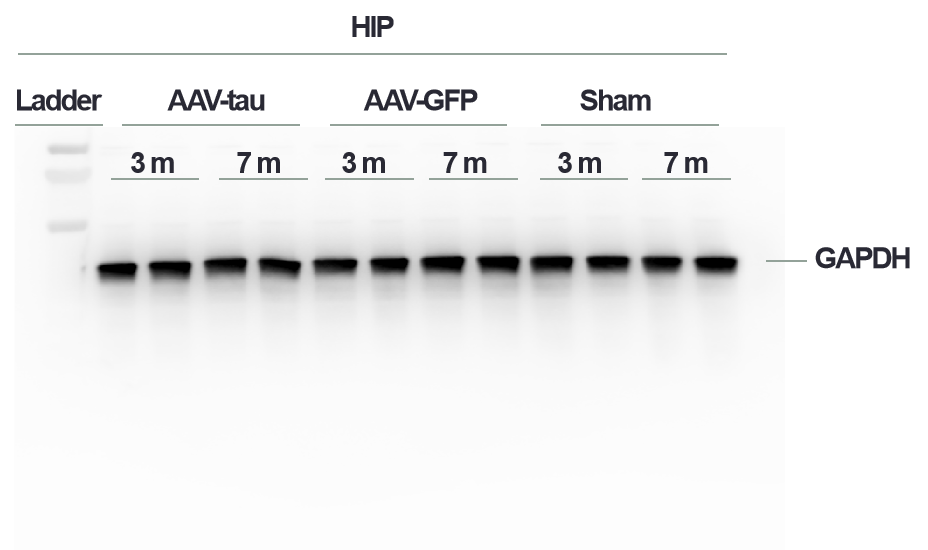
**

**
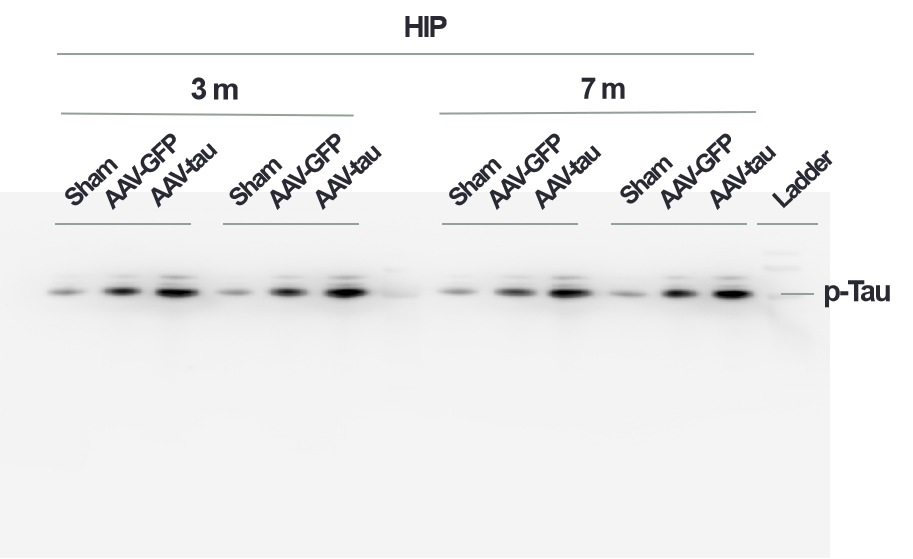

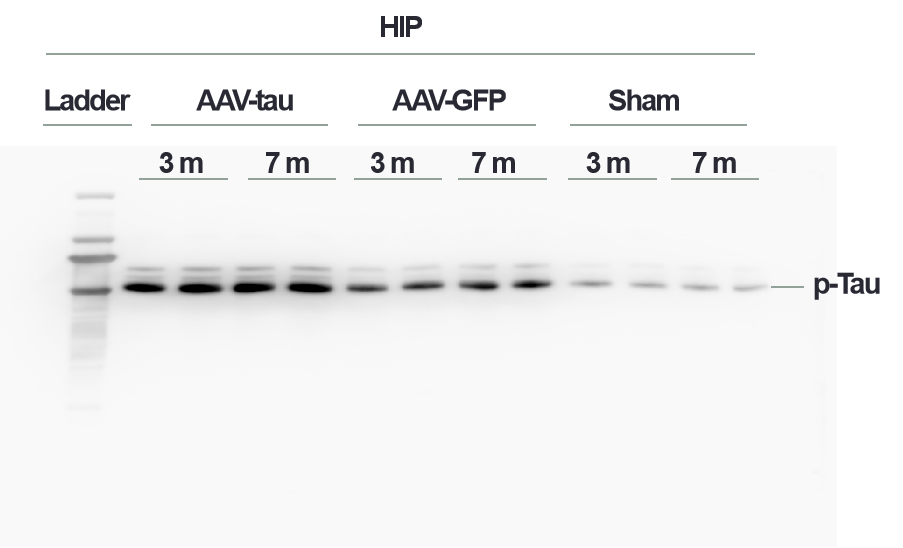
**


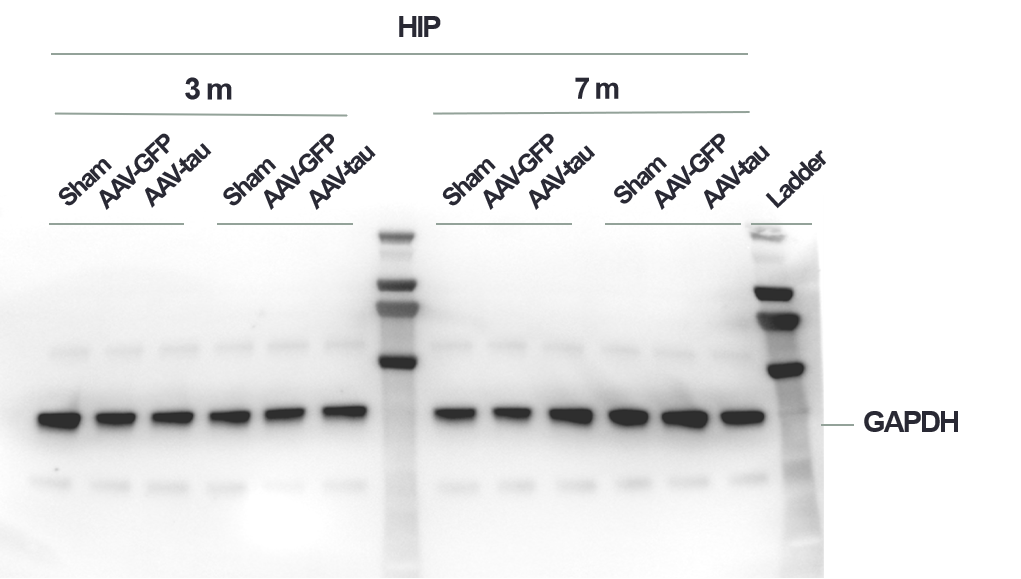

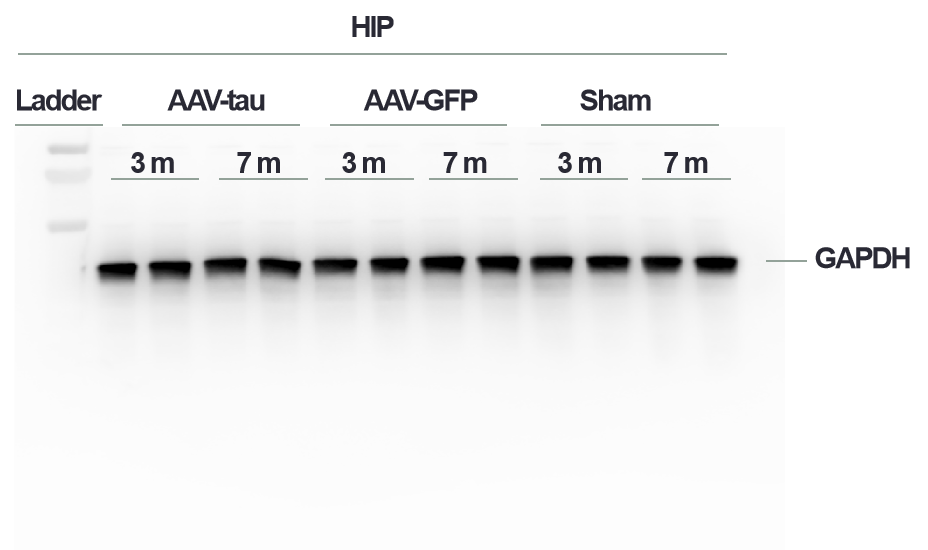


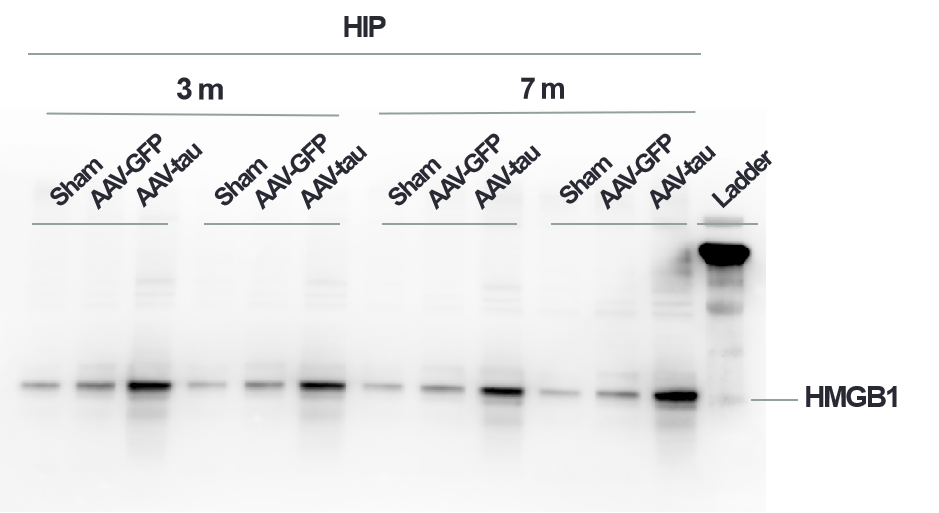

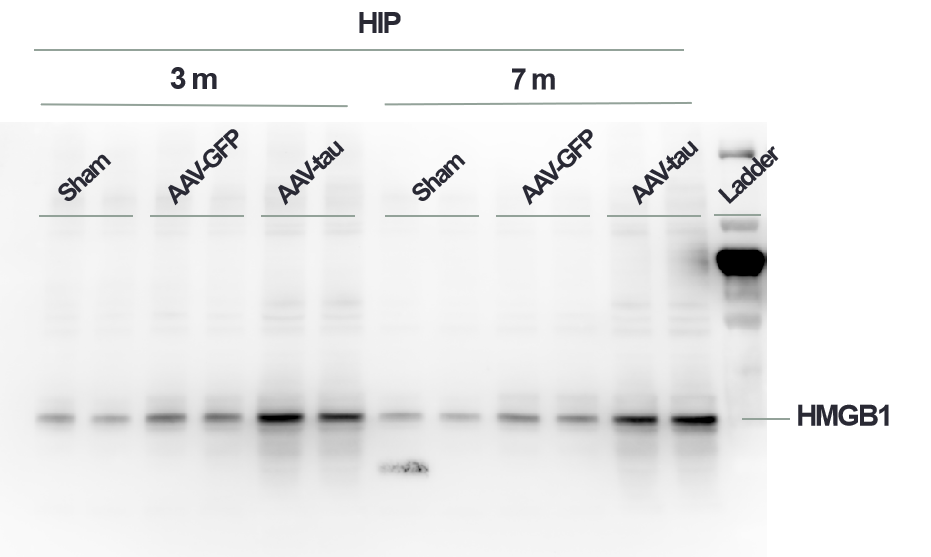

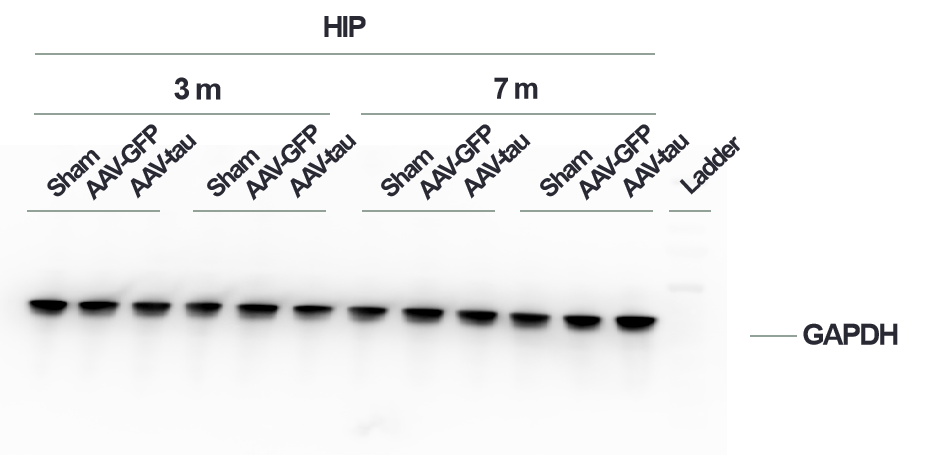

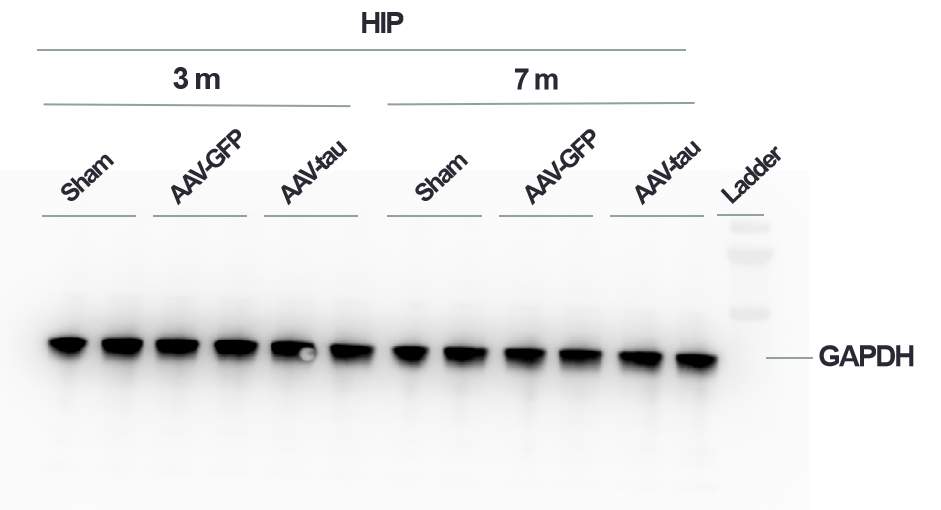


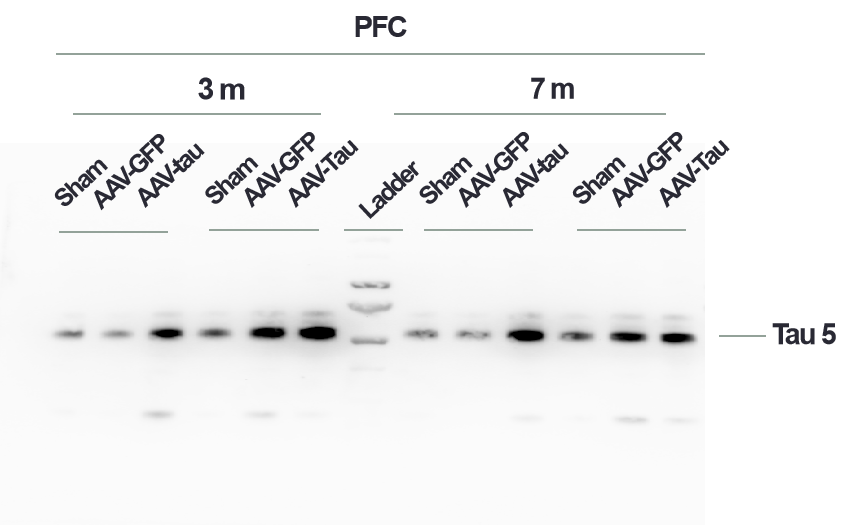

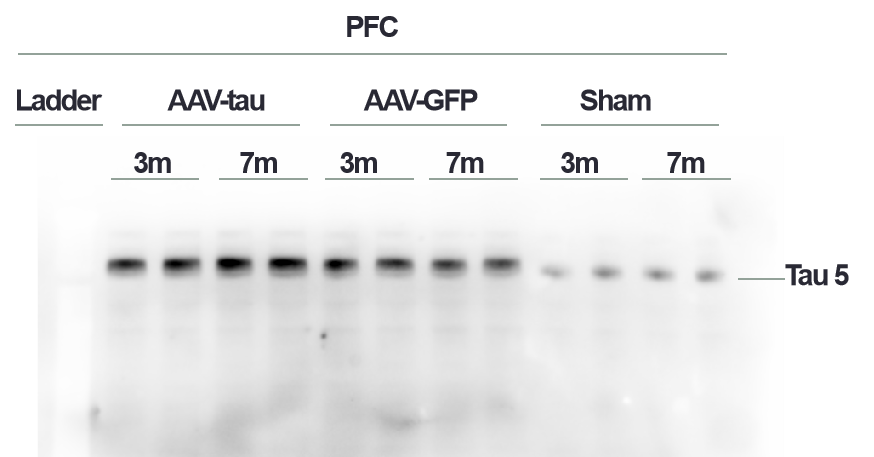


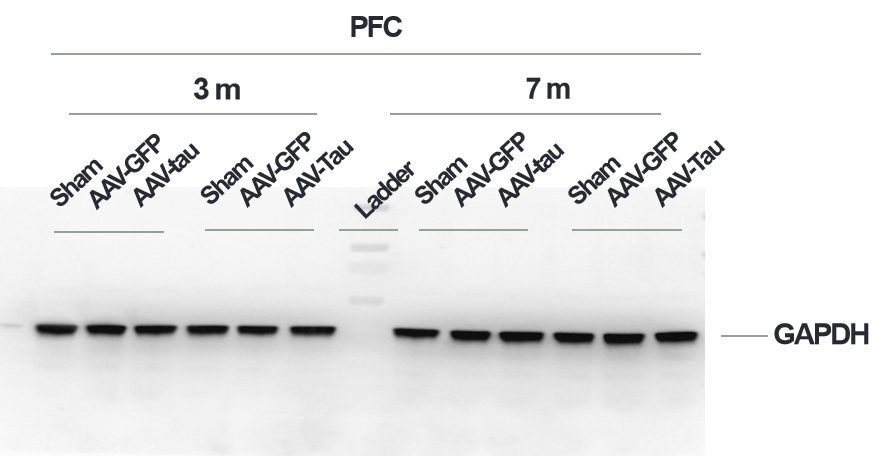

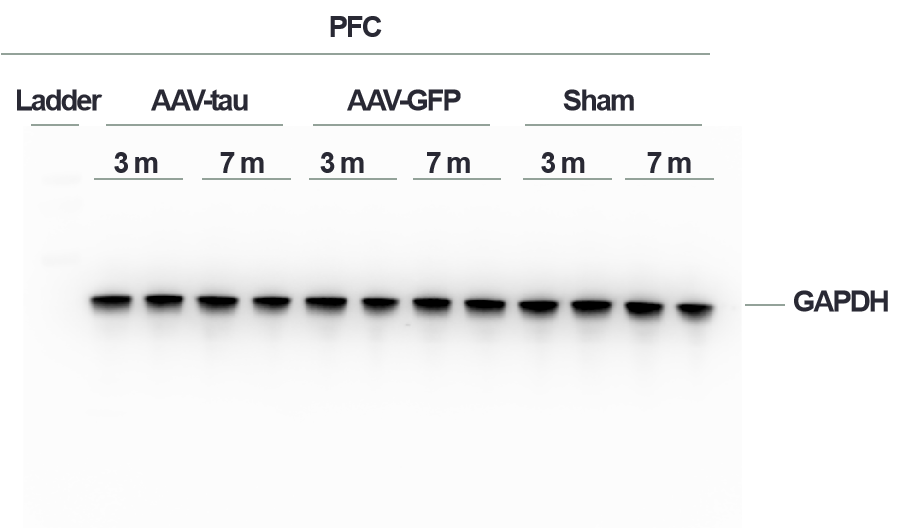


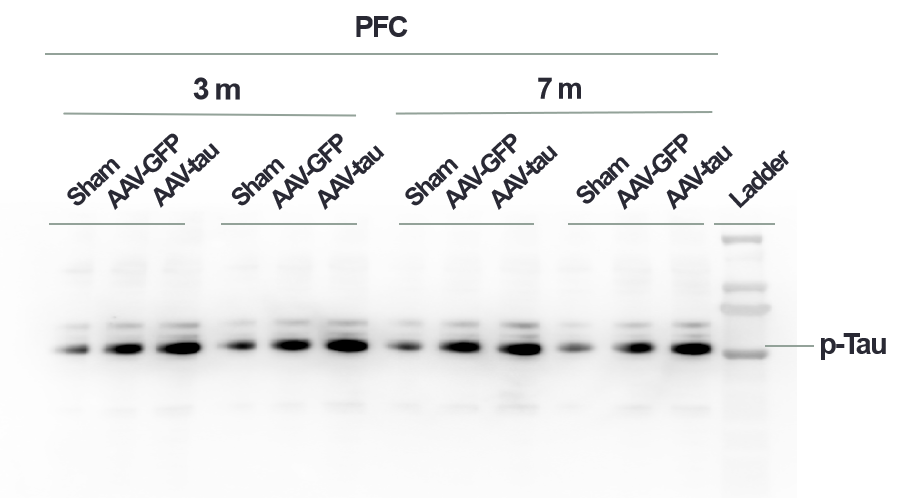

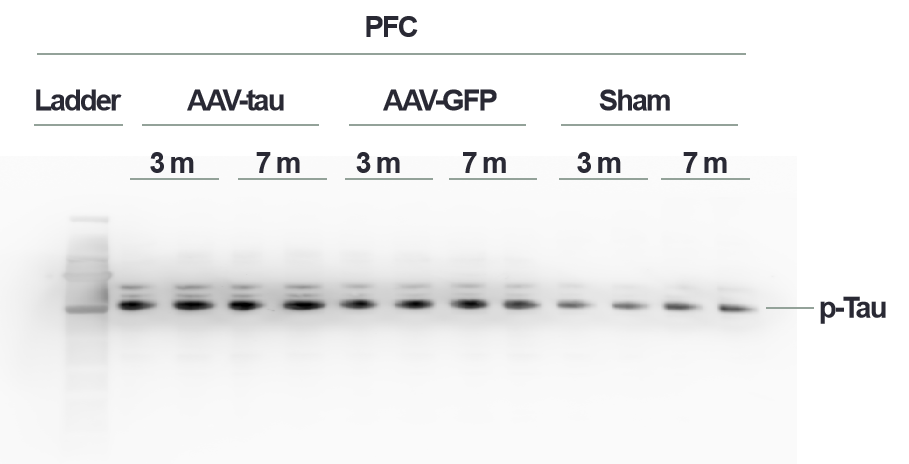


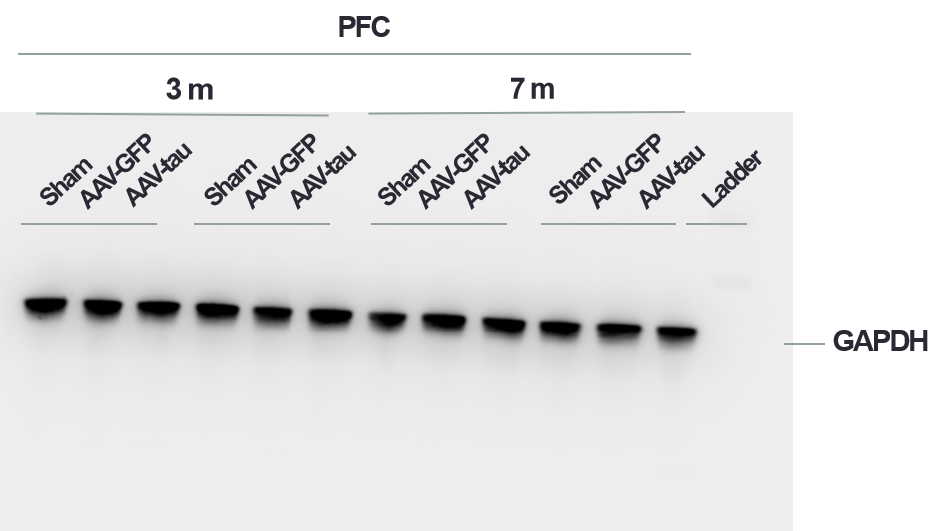

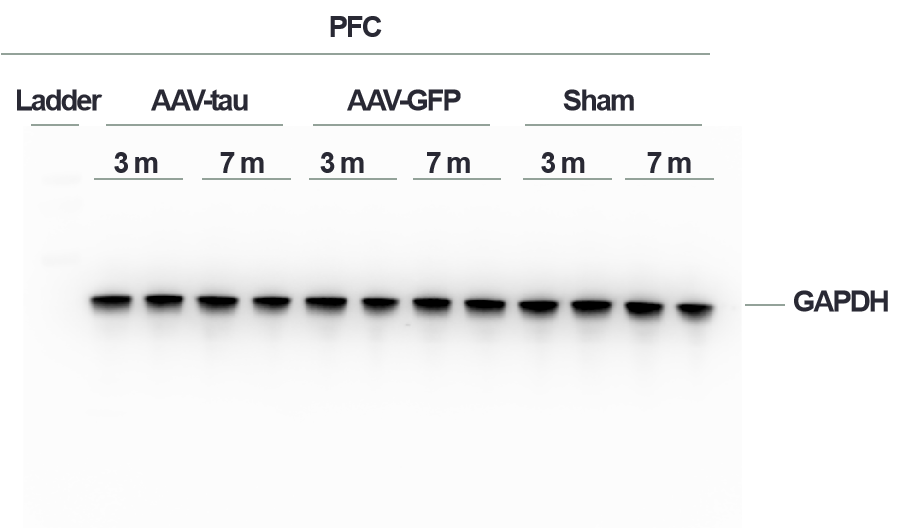


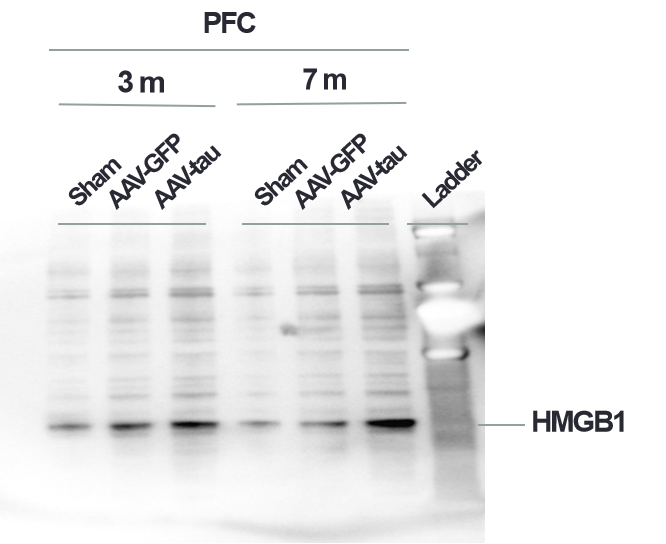

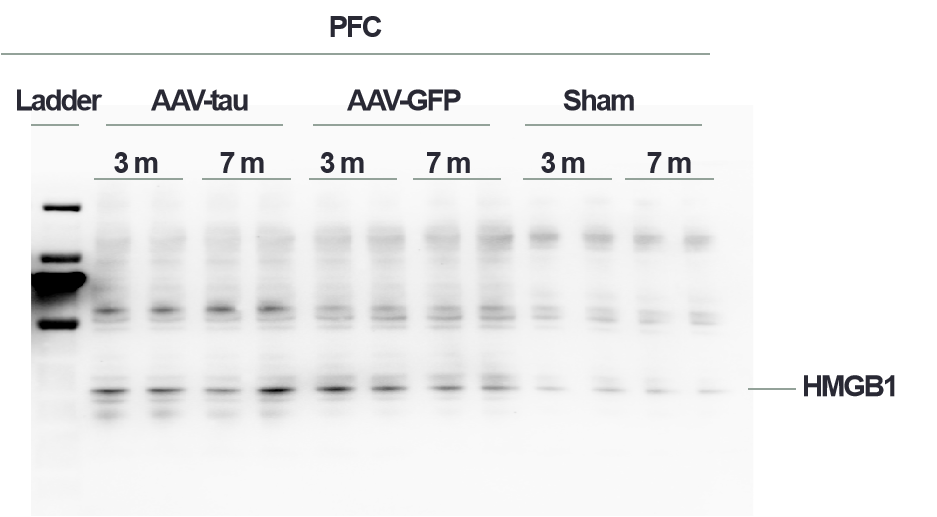


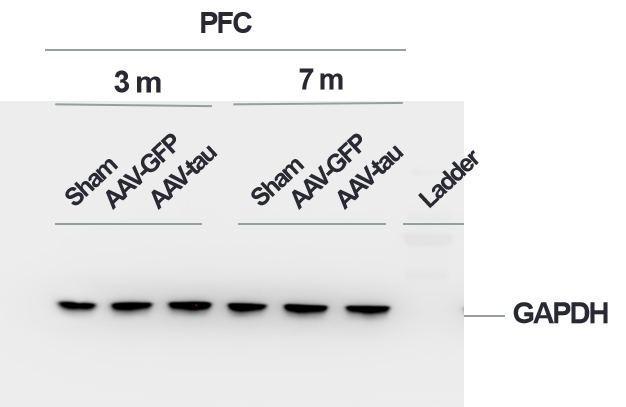

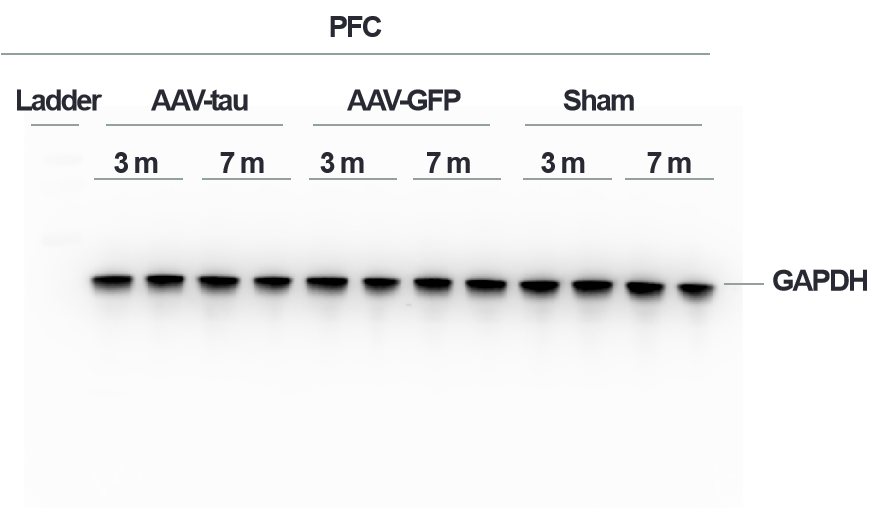


**Full blot images of Fig. 4C, E**

**
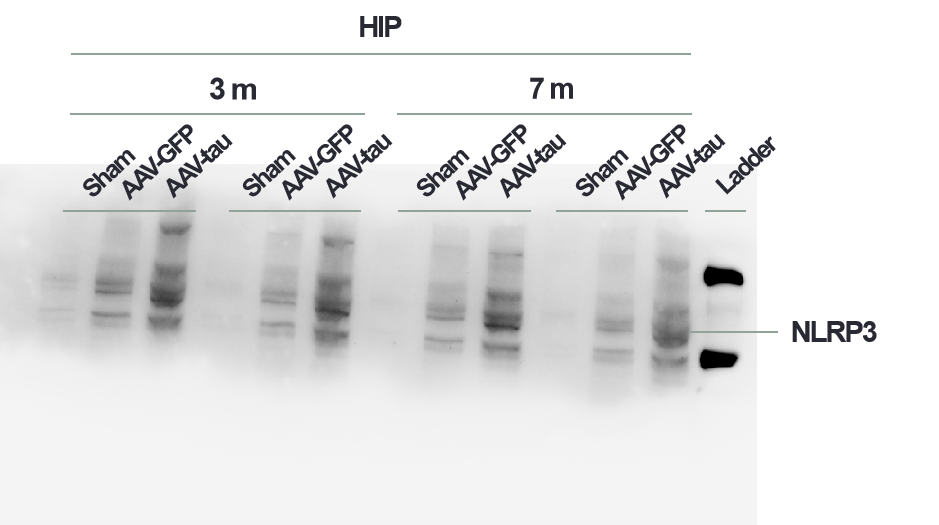

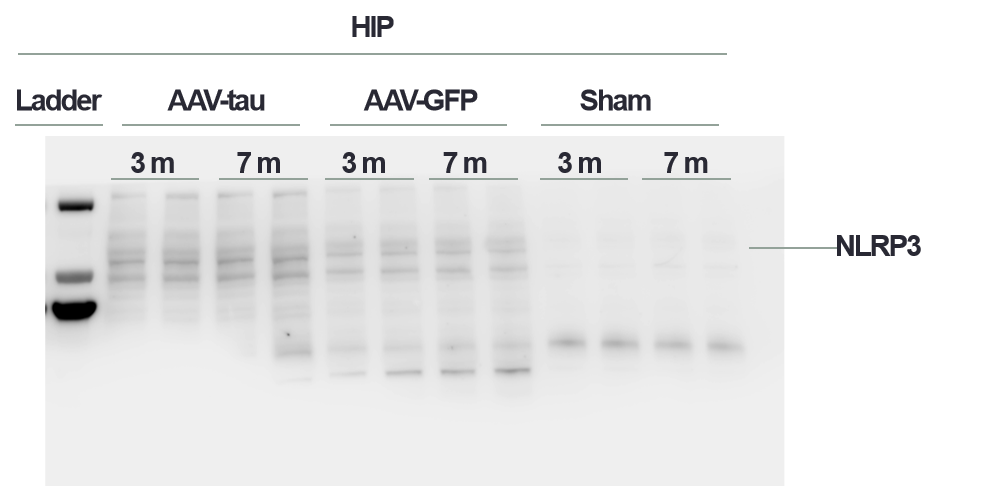
**

**
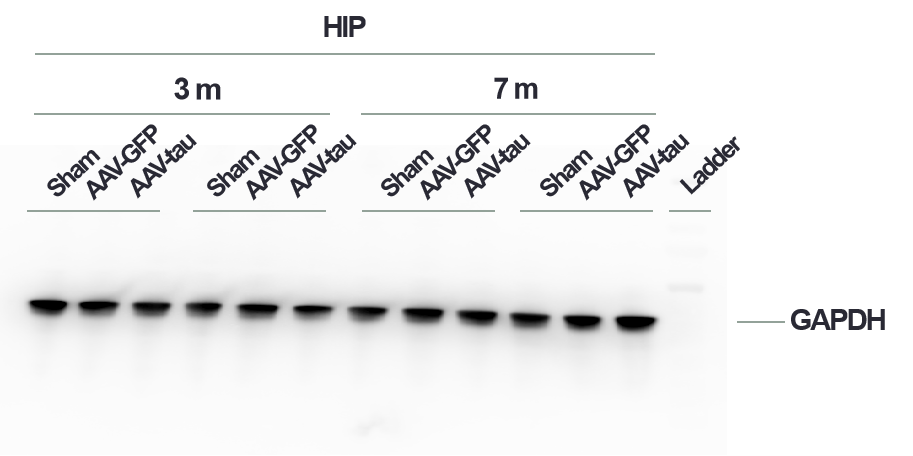

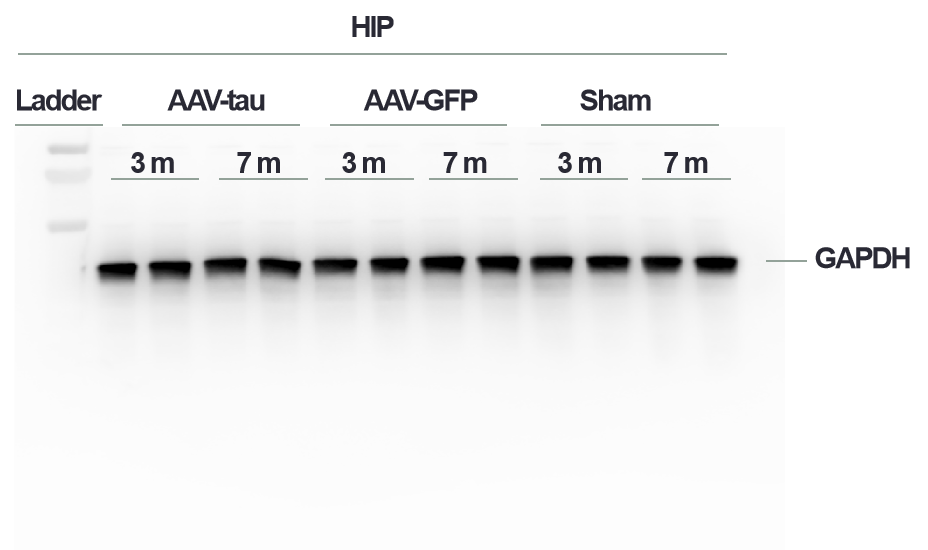
**

**
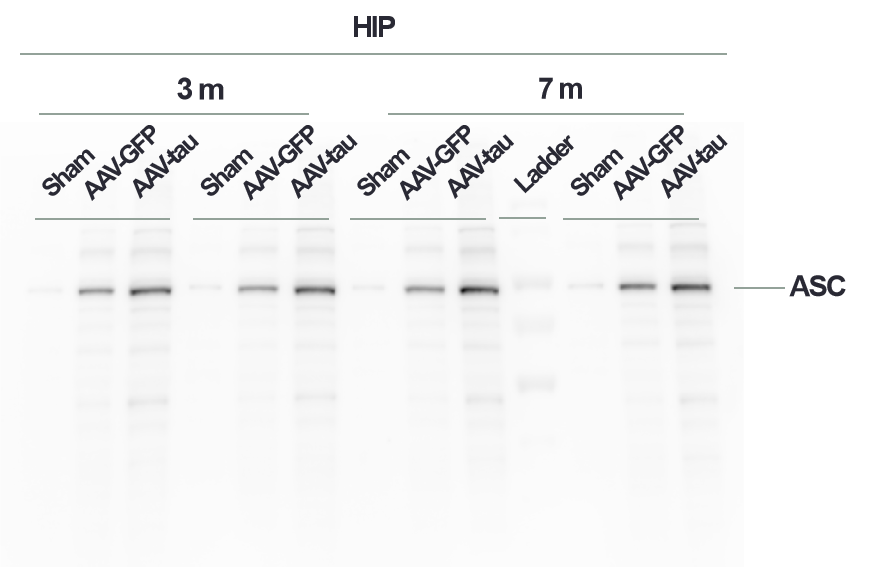

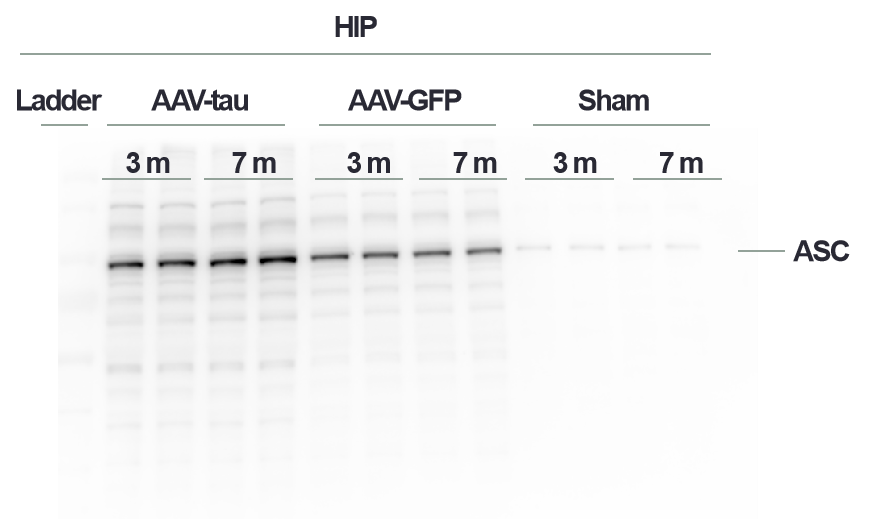
**


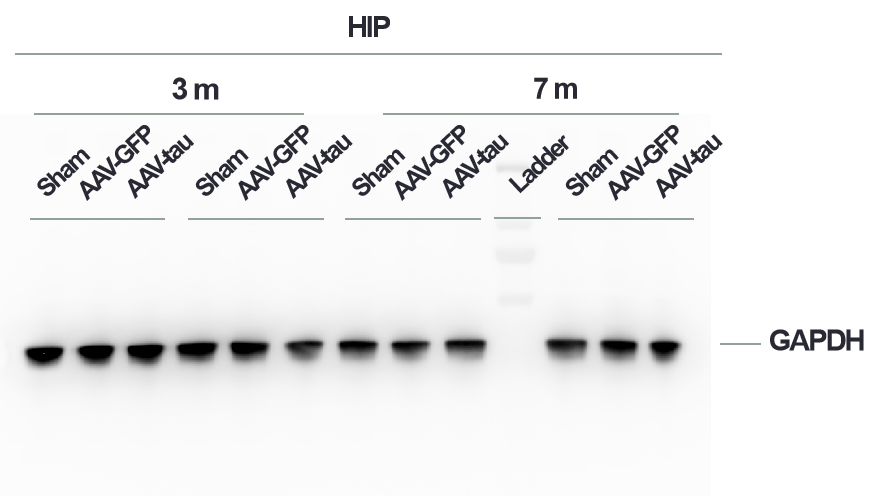

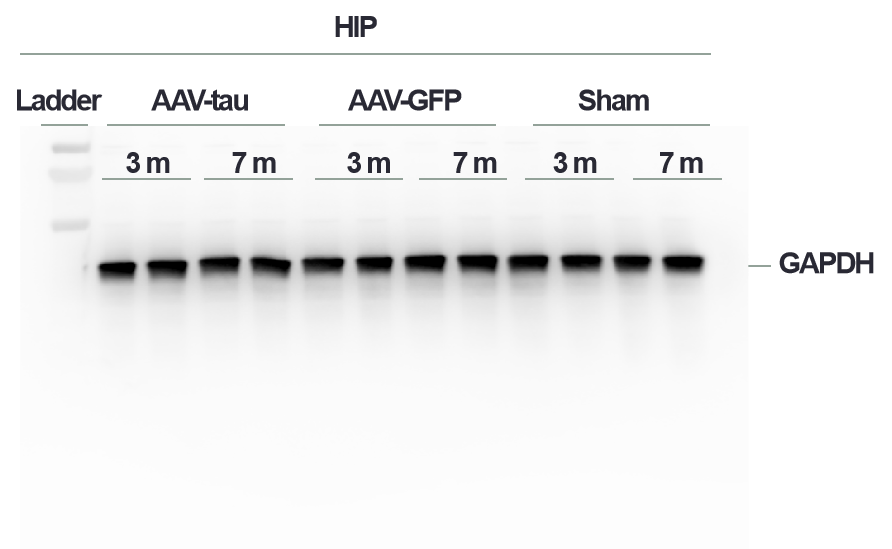


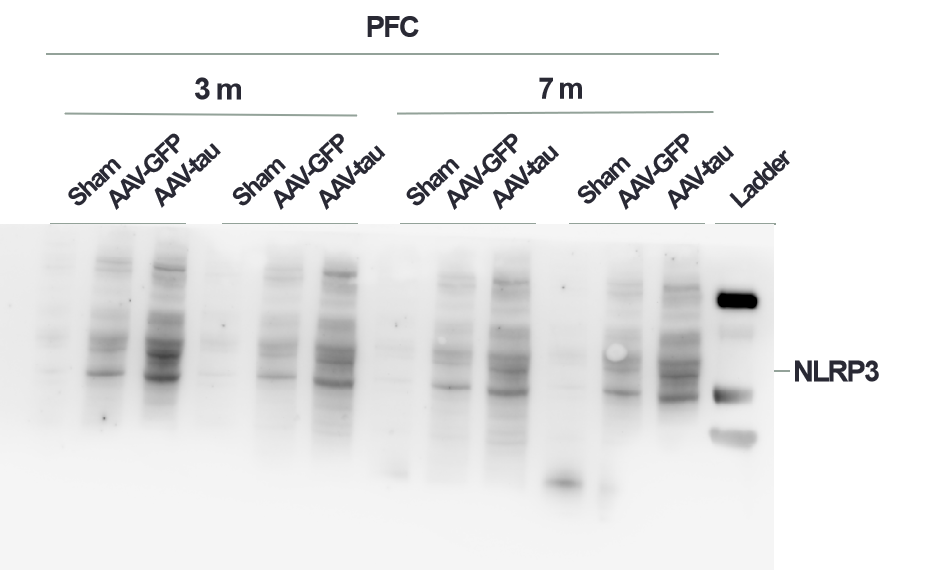

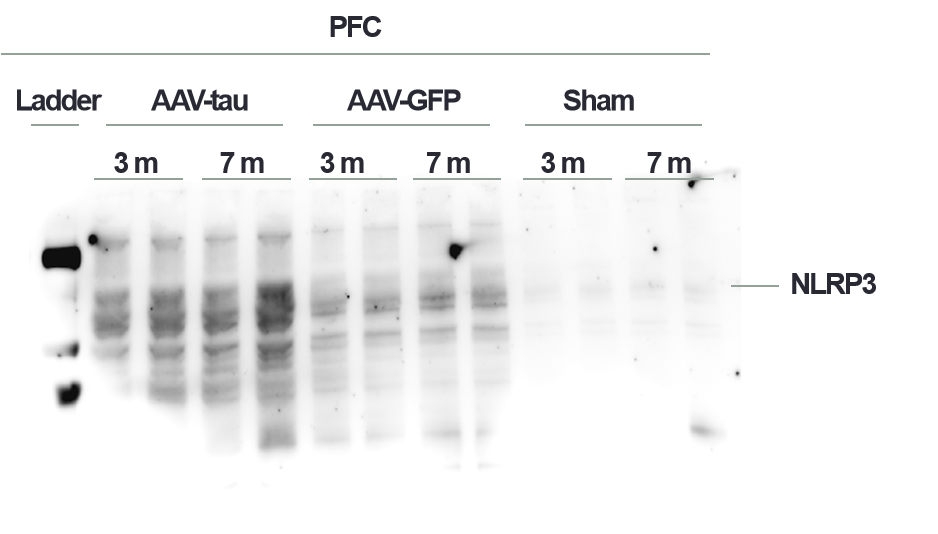


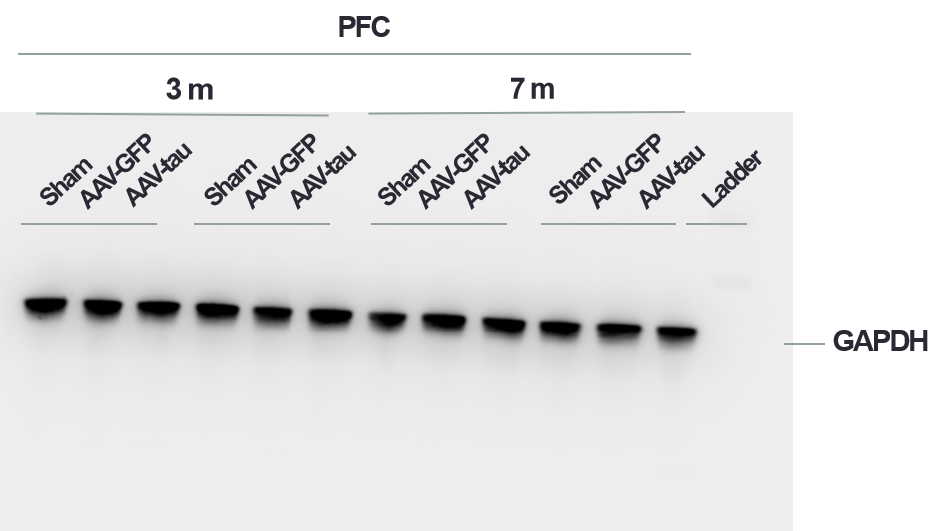

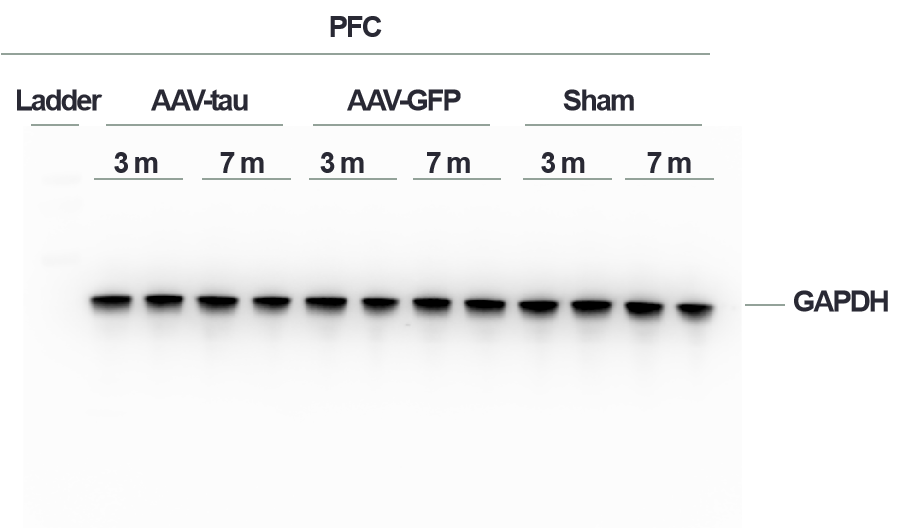


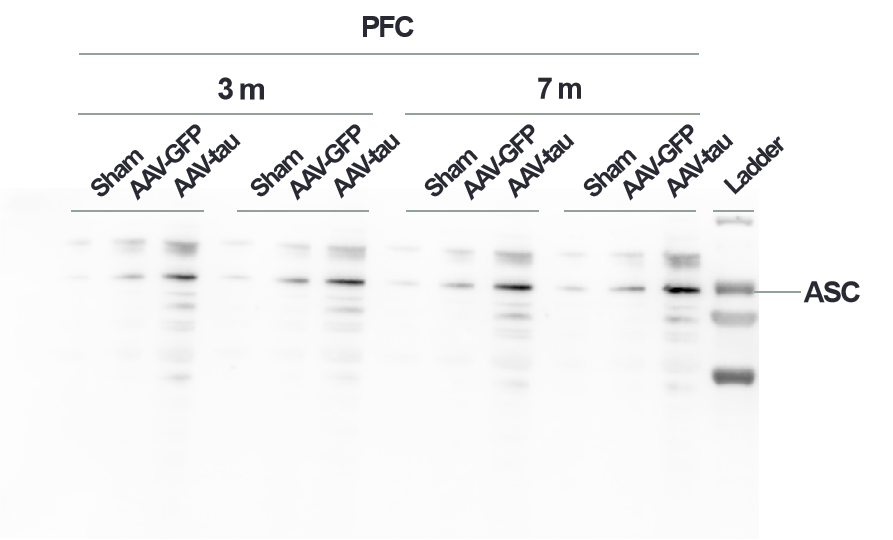

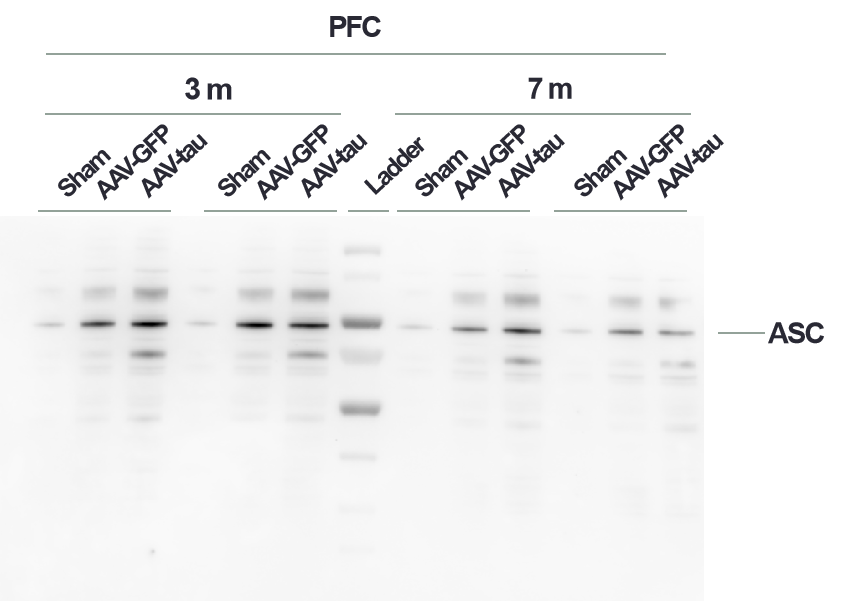


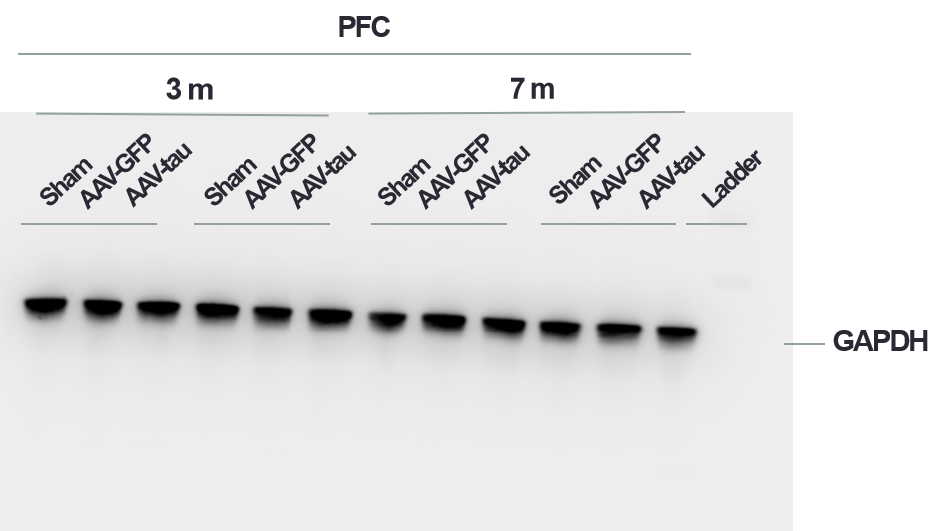

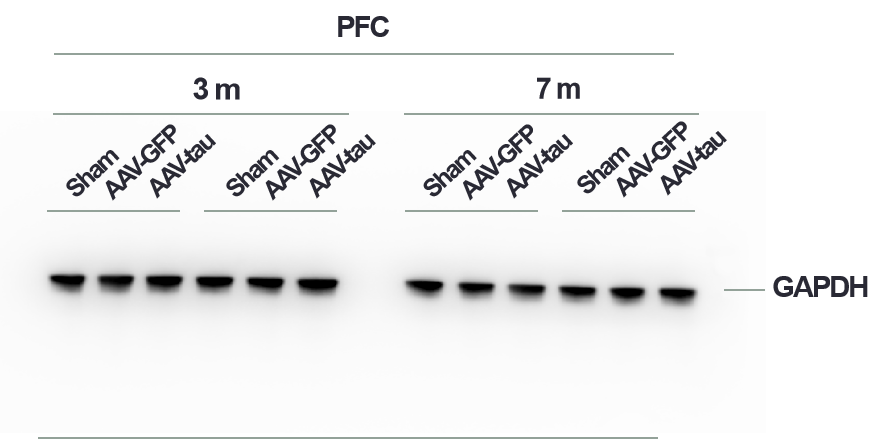


**Full blot images of Fig. 5C, D**

**
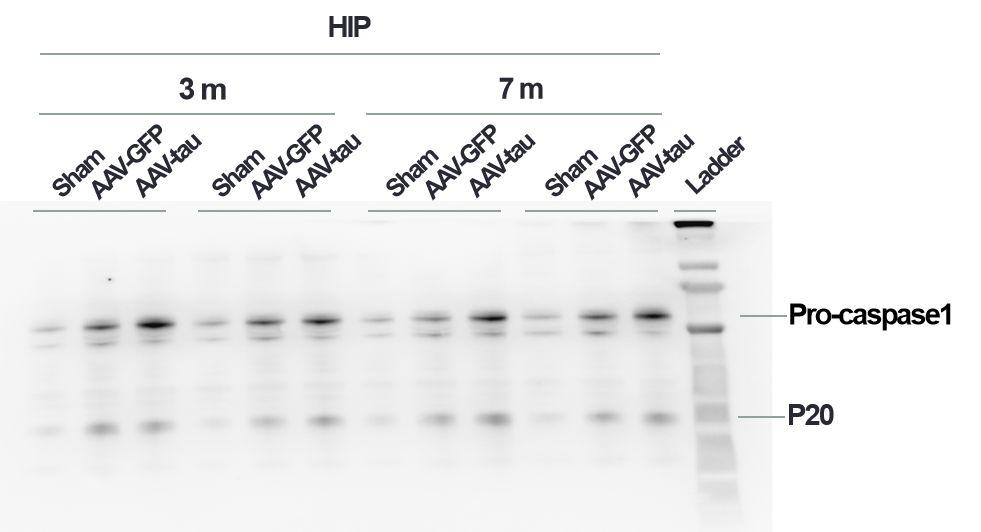

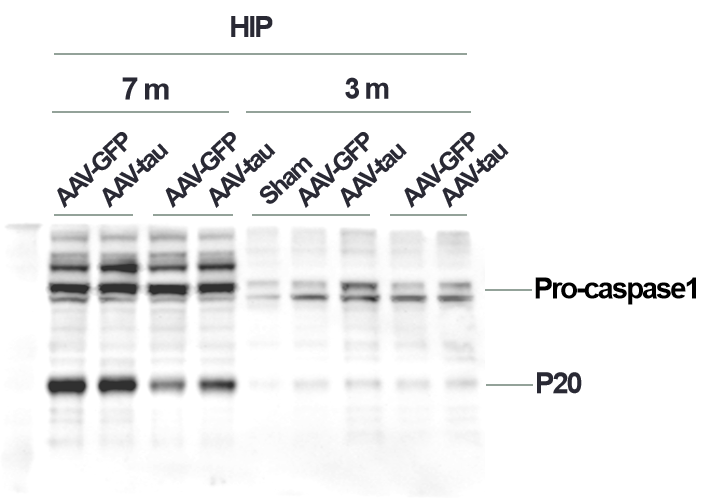

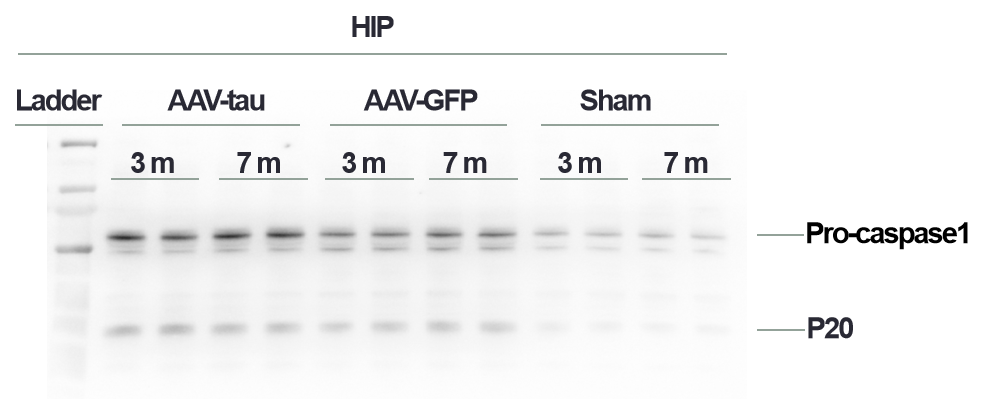
**


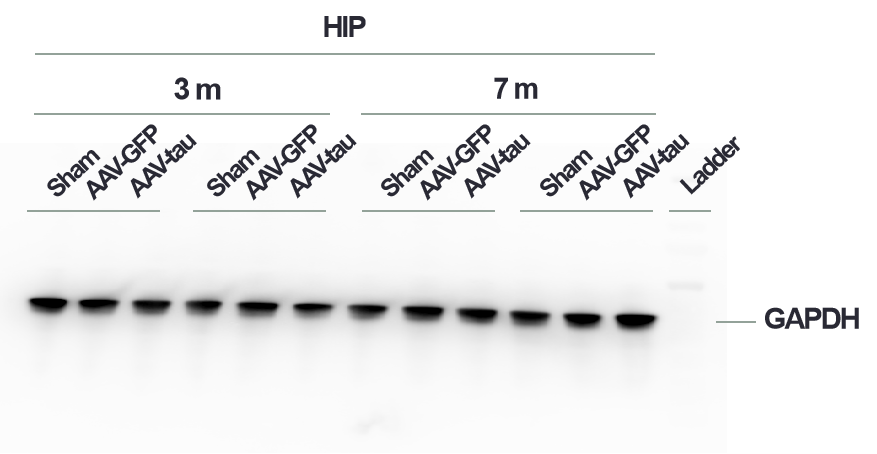

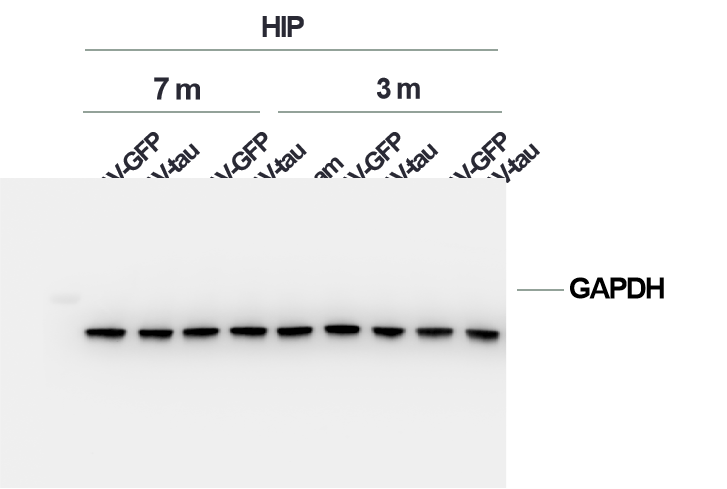

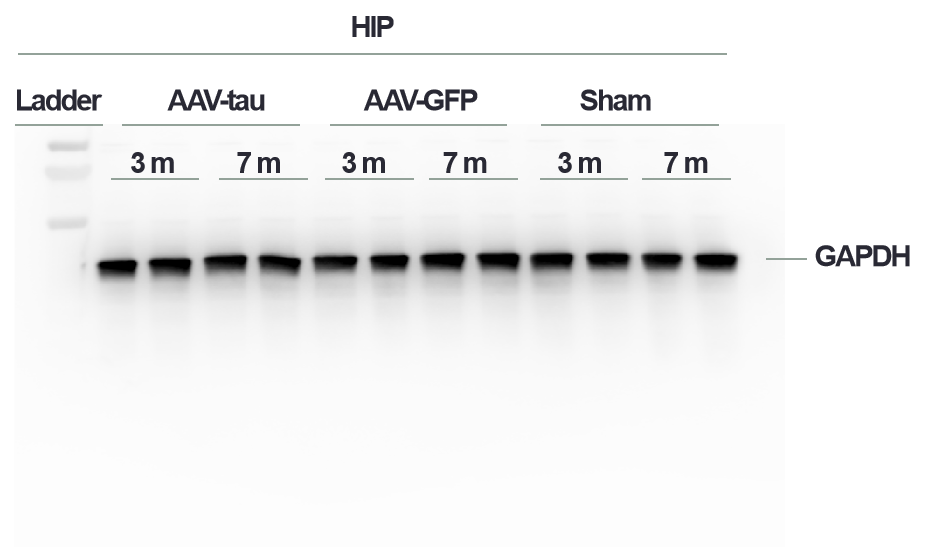


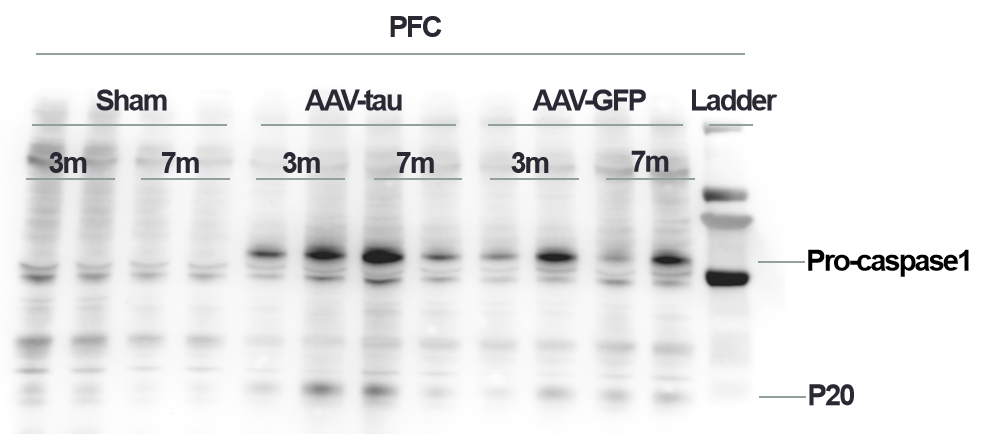

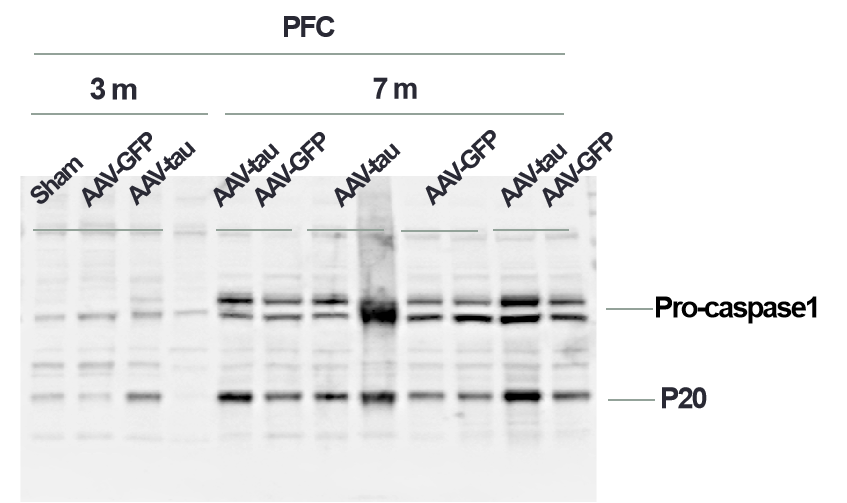

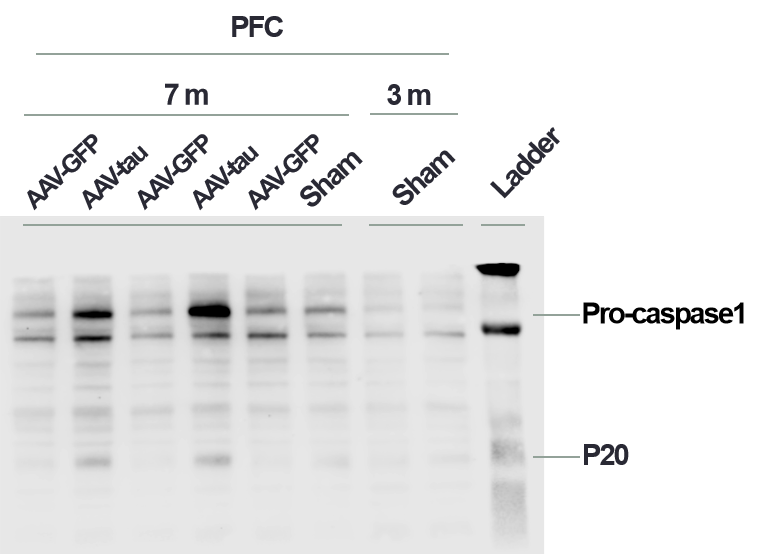


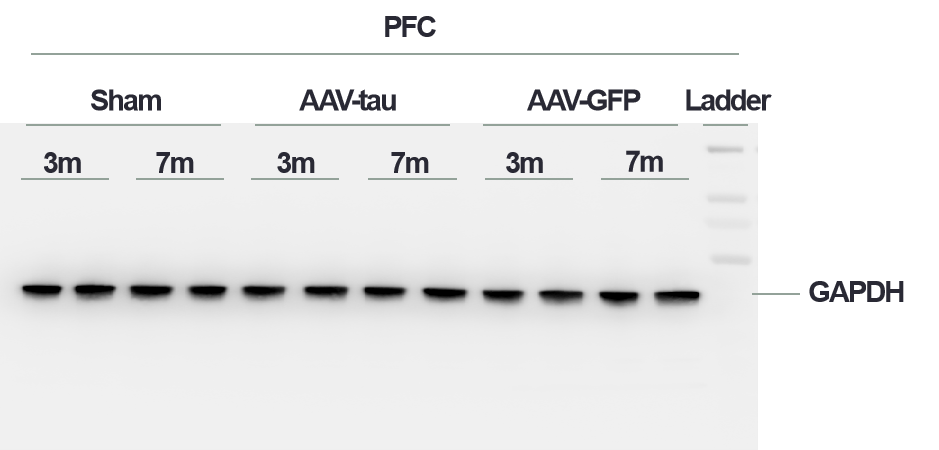

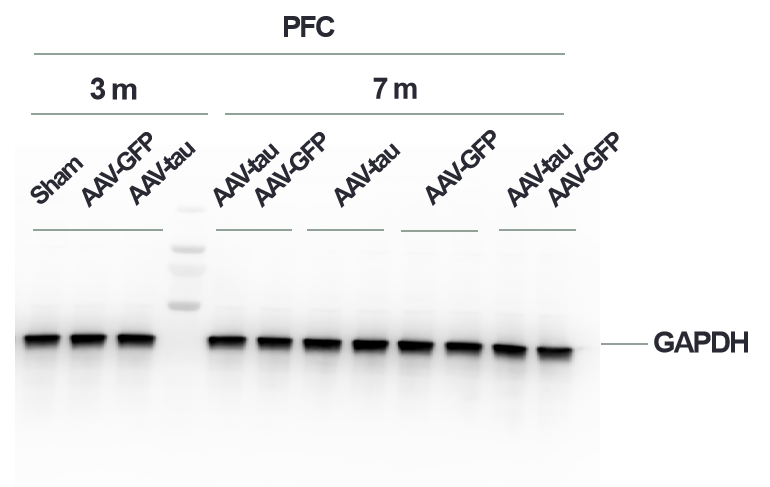

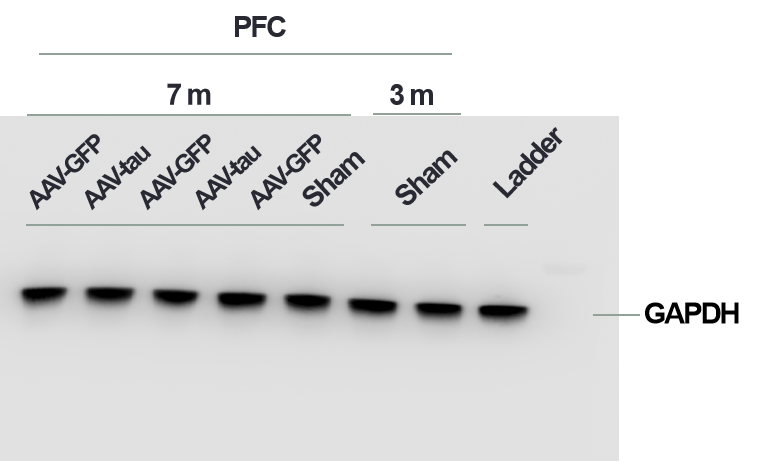

Supplement: Supplementary file 1 [file Data_Sheet_1.docx]
